# Whole-Genome Sequencing of 5-Hydroxymethylcytosine at Base Resolution by Bisulfite-Free Single-Step Deamination with Engineered Cytosine Deaminase

**DOI:** 10.1021/acscentsci.3c01131

**Published:** 2023-11-30

**Authors:** Neng-Bin Xie, Min Wang, Wei Chen, Tong-Tong Ji, Xia Guo, Fang-Yin Gang, Ya-Feng Wang, Yu-Qi Feng, Yuan Liang, Weimin Ci, Bi-Feng Yuan

**Affiliations:** † Department of Occupational and Environmental Health, School of Public Health, Wuhan University, Wuhan 430071, China; ‡ Research Center of Public Health, Renmin Hospital of Wuhan University, Wuhan University, Wuhan 430060, China; § College of Chemistry and Molecular Sciences, Wuhan University, Wuhan 430072, China; ∥ Department of Laboratory Medicine, Zhongnan Hospital of Wuhan University, Wuhan University, Wuhan 430071, China; ⊥ Key Laboratory of Genomics and Precision Medicine, and China National Center for Bioinformation, Beijing Institute of Genomics, Chinese Academy of Sciences, Beijing 100101, China; # University of Chinese Academy of Sciences, Beijing 100049, China

## Abstract

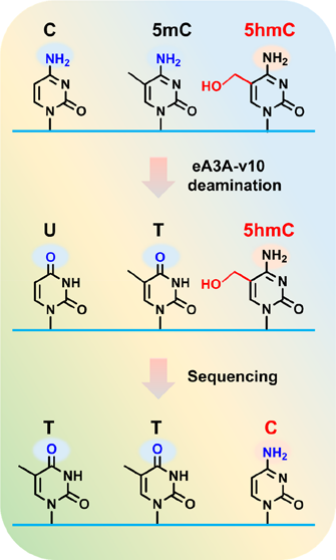

The epigenetic modification 5-hydroxymethylcytosine (5hmC) plays a crucial role in the regulation of gene expression. Although some methods have been developed to detect 5hmC, direct genome-wide mapping of 5hmC at base resolution is still highly desirable. Herein, we proposed a single-step deamination sequencing (SSD-seq) method, designed to precisely map 5hmC across the genome at single-base resolution. SSD-seq takes advantage of a screened engineered human apolipoprotein B mRNA-editing catalytic polypeptide-like 3A (A3A) protein, known as eA3A-v10, to selectively deaminate cytosine (C) and 5-methylcytosine (5mC) but not 5hmC. During sequencing, the deaminated C and 5mC are converted to uracil (U) and thymine (T), read as T in the sequencing data. However, 5hmC remains unaffected by eA3A-v10 and is read as C during sequencing. Consequently, the presence of C in the sequence reads indicates the original 5hmC. We applied SSD-seq to generate a base-resolution map of 5hmC in human lung tissue. Our findings revealed that 5hmC was predominantly localized to CpG dinucleotides. Furthermore, the base-resolution map of 5hmC generated by SSD-seq demonstrated a strong correlation with prior ACE-seq results. The advantages of SSD-seq are its single-step process, absence of bisulfite treatment or DNA glycosylation, cost effectiveness, and ability to detect and quantify 5hmC directly at single-base resolution.

## Abstract 2

A single-step deamination sequencing method was developed for genome-wide mapping of 5hmC at single-base resolution.

## Introduction

DNA cytosine methylation (5-methylcytosine, 5mC) is a predominant epigenetic modification that plays critical roles in a variety of biological and pathological processes in mammals.^1,2^ 5-Hydroxymethylcytosine (5hmC), first discovered in mammalian genomes in 2009, is now viewed as the “sixth base” of DNA.^3^ It has been demonstrated that the ten–eleven translocation (TET) family proteins can catalyze the sequential oxidation of 5mC to 5hmC, 5-formylcytosine (5fC), and 5-carboxycytosine (5caC).^4^ 5fC and 5caC can be converted to unmodified cytosines by a base excision repair pathway or through direct deformylation or decarboxylation,^5−8^ which constitutes the active DNA demethylation pathway in mammals.^9^ Beyond being an “intermediate” in 5mC oxidation, 5hmC is also a stable epigenetic modification occurring in mammalian genomes.^10^ Increasing lines of evidence suggest that 5hmC directly participates in the regulation of gene expression in both physiological and pathological states.^11−13^

Genome-wide detection of 5hmC is required to improve our understanding of 5hmC and its role in the modulation of gene expression as well as in other biological and pathological processes.^14−16^ Some methods have been developed to detect 5hmC in genomic DNA, including liquid chromatography or capillary electrophoresis with mass spectrometry (LC–MS or CE–MS) analysis.^17,18^ These methods involve the enzymatic digestion of genomic DNA into nucleosides, allowing for the quantitative measurement of the 5hmC level. However, they do not provide precise site-specific information about 5hmC in the genome. To achieve genome-wide mapping of 5hmC, affinity enrichment followed by sequencing methods has been established.^19−21^ However, these approaches have limitations in terms of resolution, typically providing a resolution range of 200–500 bp and lacking single-base resolution mapping capability.^22^ To overcome these limitations, oxidative bisulfite sequencing (oxBS-seq)^23^ and TET-assisted bisulfite sequencing (TAB-seq)^24^ methods have been developed. These techniques enable the detection of 5hmC at single-base resolution. However, it is worth noting that bisulfite treatment, a crucial step in these methods, can lead to significant degradation of input DNA by as much as 99%.^25^ Alternatively, the analysis of 5hmC can be performed using single-molecule, real-time (SMRT) and nanopore sequencing technologies.^26−29^ However, these methods have a relatively high false-positive rate in mapping modified nucleobases.^30,31^

Recent studies have shown that the wild-type APOBEC3A (apolipoprotein B mRNA-editing catalytic polypeptide-like 3A or wtA3A) protein exhibits efficient deamination activity toward C and 5mC.^32^ It has also been observed that wtA3A can deaminate 5hmC to a lesser extent but does not show deamination activity toward glycosylated 5hmC (β-glucosyl-5-hydroxymethyl-2′-deoxycytidine, 5gmC).^32,33^ With this property of wtA3A, we and others developed A3A-mediated deamination sequencing (AMD-seq)^33^ and A3A-coupled epigenetic sequencing (ACE-seq)^34^ for mapping 5hmC in DNA at base resolution. However, pretreatment of DNA with *β-*glucosyltransferase (β-GT) to convert 5hmC to 5gmC is indispensable in these methods. The comparison between TET-assisted pyridine borane sequencing (TAPS) and β-glucosyltransferase blocking TET-assisted pyridine borane sequencing (TAPSβ) also allows the detection of 5hmC at single-base resolution.^35^ This strategy, however, is indirect and requires the TET-mediated oxidation of 5mC, glycosylation of 5hmC, and comparison of the sequencing results from two approaches. We recently engineered the wtA3A protein and proposed an engineered deaminase-mediated sequencing (EDM-seq) for the detection of 5hmC in DNA at single-base resolution.^36^ It should be noted that the use of two engineered A3A proteins in EDM-seq poses a challenge for the genome-wide mapping of 5hmC. EDM-seq is suitable for the detection of 5hmC at individual sites in DNA rather than for genome-wide mapping.

Herein, we conducted extensive engineering of A3A proteins derived from the wtA3A protein. Through screening, we identified a specific engineered A3A variant, referred to as eA3A-v10. This variant demonstrated effective deamination activity toward C and 5mC while exhibiting no deamination activity toward 5hmC in different sequence contexts of DNA. Based on the property of eA3A-v10, we developed a novel sequencing method called single-step deamination sequencing (SSD-seq). This method allows for genome-wide mapping of 5hmC at single-base resolution in a direct and efficient manner.

## Results and Discussion

### Principle of the Single-Step Deamination Sequencing (SSD-seq)

A previous study showed that the wtA3A protein could deaminate C and 5mC to generate U and T, respectively.^32^ However, the deamination activity of wtA3A toward 5hmC is weaker compared to that toward C and 5mC.^32^ As a result, when treated with wtA3A, C and 5mC are read as T while 5hmC is partially read as C and partially read as T in sequencing (Figure S1). This poses a challenge in readily differentiating 5hmC from C and 5mC. To overcome this limitation, we previously engineered the wtA3A protein to enhance its selectivity and developed the EDM-seq method.^36^ This method utilized two engineered A3A proteins for the site-specific detection of 5hmC in DNA. However, the use of two engineered A3A proteins in EDM-seq makes the genome-wide mapping of 5hmC challenging. This is primarily due to the complex sequencing readouts, which can make it extremely difficult to accurately map the reads to reference genomes (Figure S2). Consequently, EDM-seq is suitable only for the detection of 5hmC at individual sites in DNA and does not meet the requirements for the genome-wide mapping of 5hmC.

**Figure 1:**
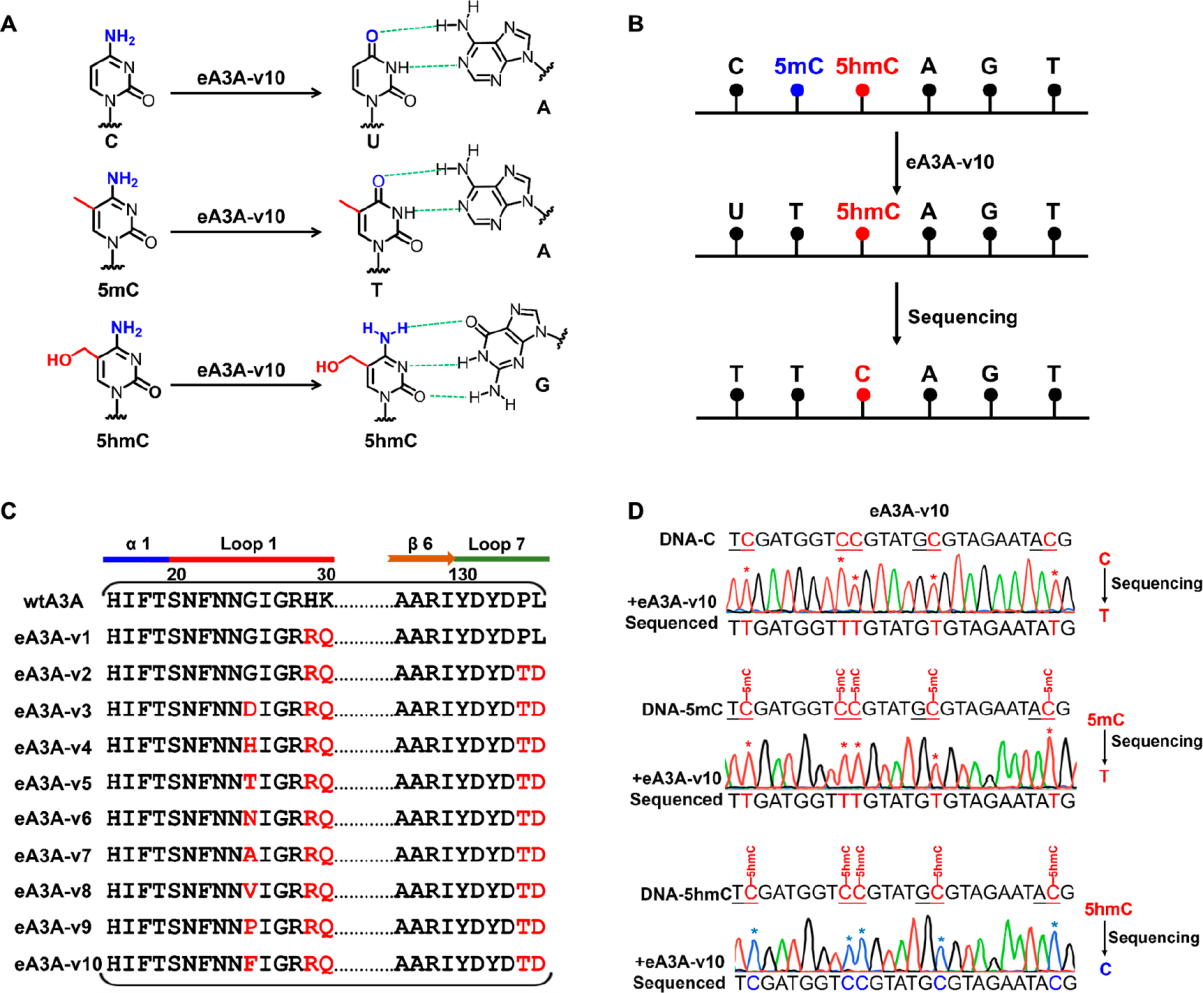
Principle of SSD-seq. (A) C and 5mC can be deaminated by eA3A-v10 to form U and T, respectively, both of which base pair with A. 5hmC is not deaminated by eA3A-v10 and still base pairs with G. (B) In SSD-seq, C and 5mC are deaminated to form U and T, both of which are read as T during sequencing. However, 5hmC is resistant to deamination by eA3A-v10 and still reads as C during sequencing. The readouts of C from sequence reads manifest the original 5hmC sites in DNA. (C) Amino acid compositions of wtA3A and engineered A3A variants (eA3A-v1 to eA3A-v10). (D) Sanger sequencing results of DNA-C, DNA-5mC, and DNA-5hmC were obtained by SSD-seq.

In the current study, we successfully screened a single engineered A3A variant known as eA3A-v10. This variant demonstrated robust deamination activity toward C and 5mC while being inert toward 5hmC (Figure 1A). Utilizing the screened eA3A-v10, we developed a novel sequencing method called single-step deamination sequencing (SSD-seq). In SSD-seq, eA3A-v10 actively deaminates the original C and 5mC in DNA, converting them to U and T, respectively. Consequently, these deaminated bases are read as T in the sequencing results (Figure 1B). On the other hand, 5hmC is resistant to the deamination by eA3A-v10 and thus remains as it is during sequencing, being read as C (Figure 1B). As a result, the remaining C in the sequence reads precisely indicates the original 5hmC sites in DNA, providing a means for the single-base resolution detection of 5hmC (Figure 1B).

### Screening of eA3A Proteins

As for the development of SSD-seq, we aimed to screen a single engineered A3A variant that could deaminate C and 5mC but not 5hmC. According to the crystal structure of wtA3A, the amino acid residues around loop 1 (residues 20 to 31) and loop 7 (residues 130 to 135) have important roles in the intrinsic substrate preference.^37−39^ Specifically, the amino acid residues T31 (T, threonine) in loop 1 and Y130 (Y, tyrosine) in loop 7 have been shown to play key roles in positioning cytosine by directly interacting with the pyrimidine ring.^39^ Additionally, other amino acids such as G25 (G, glycine), H29 (H, histidine), K30 (K, lysine) in loop 1, and P134 (P, proline) and L135 (L, leucine) in loop 7 have been found to influence the substrate preference of wtA3A toward cytosine.^36,38^ Therefore, we engineered a series of A3A variants (eA3A-v1 to eA3A-v10) by changing a subset of residues around the key amino acids in loops 1 and 7 of wtA3A (Figure 1C). Previous studies demonstrated that the neighboring 5′ nucleobase of cytosine could influence the deamination activity of wtA3A toward cytosine.^38^ The deamination of C, 5mC, and 5hmC by engineered A3A proteins was evaluated using three kinds of dsDNA (DNA-C, DNA-5mC, and DNA-5hmC; Table S1), with C, 5mC, and 5hmC located in different sequence contexts of GC, AC, TC, and CC sites.

eA3A-v1 was obtained with H29R (R, arginine) and K30Q (Q, glutamine) mutations in loop 1 of wtA3A (Figure S3A). The sequencing results showed that eA3A-v1 could readily deaminate C and 5mC but also partially deaminate 5hmC in TC and CC sites (Figure S3B). eA3A-v2 was generated with P134T and L135D (D, aspartic acid) mutations in loop 7 of eA3A-v1 (Figure S4A). The sequencing results showed that eA3A-v2 had excellent deamination activity toward C but could only partially deaminate 5mC and showed no deamination activity toward 5hmC (Figure S4B). Thus, eA3A-v1 and eA3A-v2 could not meet the requirement in developing SSD-seq.

It has been reported that the alteration of G25 in loop 1 of wtA3A could also affect the intrinsic substrate preference of wtA3A.^37,38,40^ On the basis of eA3A-v2, we further generated eight kinds of eA3A variants (eA3A-v3 to eA3A-v10). eA3A-v3 was generated by replacing G25 in loop 1 of eA3A-v2 with the negatively charged aspartic acid (G25D, Figure S5A). The sequencing results showed that eA3A-v3 had good deamination activity to C but only partially deaminated 5mC and showed no deamination activity toward 5hmC (Figure S5B). eA3A-v4 was produced with the G25H mutation in loop 1 of eA3A-v2 (Figure S6A). Similar to wtA3A, eA3A-v4 could readily deaminate C and 5mC, but it also showed considerable deamination activity with respect to 5hmC (Figure S6B).

eA3A-v5 and eA3A-v6 were obtained, with G25 in loop 1 of eA3A-v2 being replaced by more hydrophilic amino acids, threonine and asparagine, respectively (Figures S7A and S8A). Like eA3A-v1, eA3A-v5 and eA3A-v6 could fully deaminated C and 5mC but also showed moderate activity toward 5hmC (Figures S7B and S8B). eA3A-v7, eA3A-v8, eA3A-v9, and eA3A-v10 were generated by replacing G25 of eA3A-v2 with more hydrophobic amino acids, alanine (A), valine (V), proline (P), and phenylalanine (F), respectively (Figures S9–S12). eA3A-v7 and eA3A-v8 showed good deamination activities toward C and 5mC but also partially deaminated 5hmC (Figures S9 and S10). eA3A-v9 could fully deaminate C but only partially deaminated 5mC and showed no deamination activity toward 5hmC (Figure S11). Gratifyingly, eA3A-v10 exhibited excellent deamination activity toward C and 5mC. In the meantime, eA3A-v10 showed no deamination activity toward 5hmC in different sequence contexts of DNA (Figure 1D). We reason that the eA3A-v10 variant, with a smaller cavity between T31 and Y130, hinders the load of 5hmC due to its larger group at the C5 position. However, since C and 5mC have smaller groups at the C5 position, they can still be loaded into the catalytic center of eA3A-v10 and subsequently undergo deamination. The deamination characteristics of all of these eA3A proteins are summarized in Table S2. Since eA3A-v10 differentially deaminates C/5mC and 5hmC in DNA, it meets the requirement for the development of SSD-seq.

### Characterization of the Deamination of C, 5mC, and 5hmC by eA3A-v10

**Figure 2:**
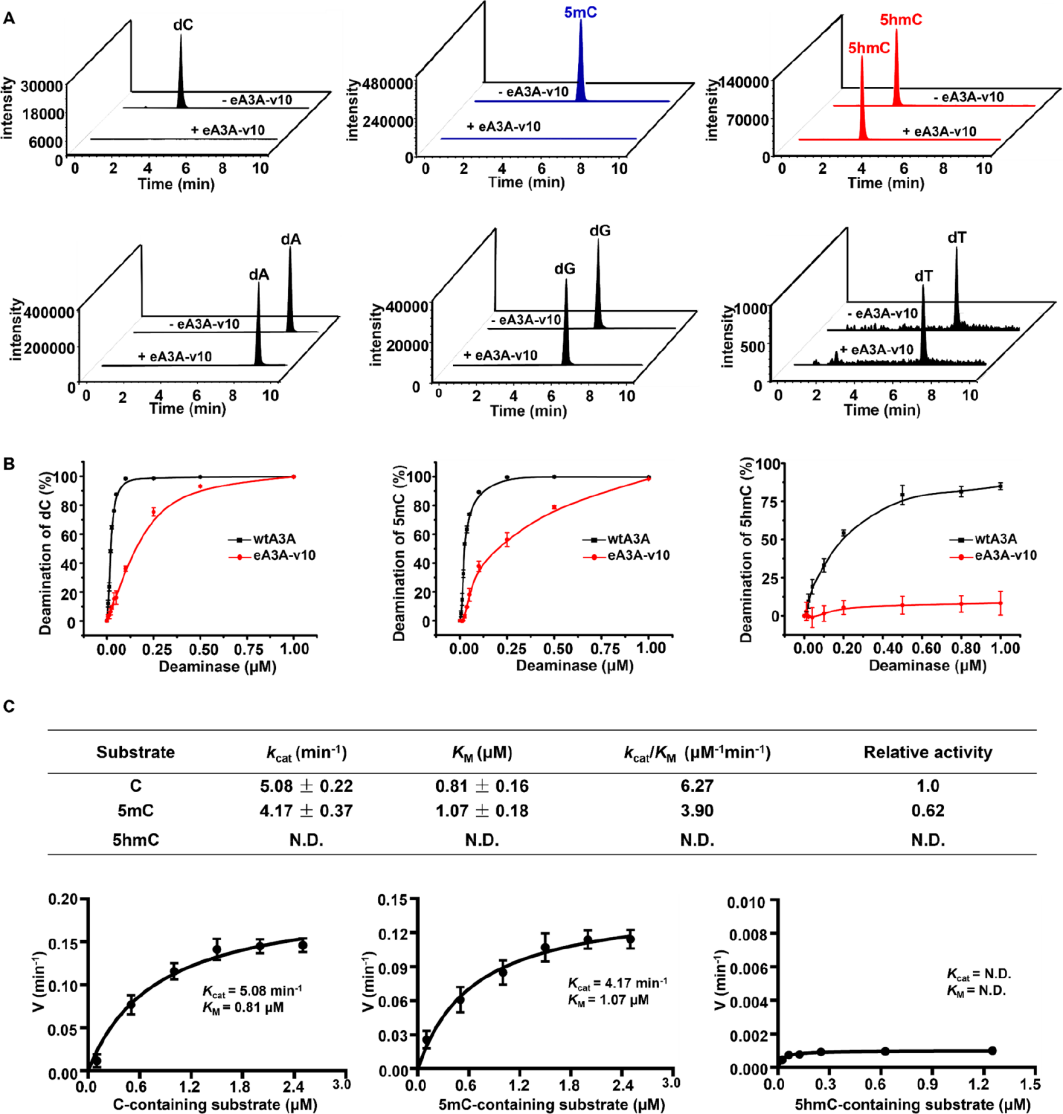
Evaluation of the deamination property of eA3A-v10 toward C, 5mC, and 5hmC by LC–MS/MS analysis. The C-containing DNA mixture (TC-C, CC-C, AC-C, and GC-C), 5mC-containing DNA mixture (TC-5mC, CC-5mC, AC-5mC, and GC-5mC), and 5hmC-containing DNA mixture (TC-5hmC, CC-5hmC, AC-5hmC, and GC-5hmC) were treated with eA3A-v10 followed by enzymatic digestion and LC–MS/MS analysis. (A) Extracted-ion chromatograms of dC (from the C-containing DNA mixture), 5mC (from the 5mC-containing DNA mixture), 5hmC (from the 5hmC-containing DNA mixture), dA (from the C-containing DNA mixture), dG (from the C-containing DNA mixture), and dT (from the C-containing DNA mixture). (B) Deamination percentages of C, 5mC, and 5hmC were treated with different concentrations of eA3A-v10 or wtA3A. (C) Kinetic constants of eA3A-v10 acting on C, 5mC, and 5hmC.

We next employed LC–MS/MS to evaluate the deamination property of eA3A-v10 toward C, 5mC, and 5hmC. Since the neighboring 5′ nucleobase of cytosine may influence the activity of deaminase, DNA strands with cytosines in different sequence contexts were used as the substrates in the evaluation, including the C-containing DNA mixture (TC-C, AC-C, GC-C, and CC-C; Table S3), 5mC-containing DNA mixture (TC-5mC, AC-5mC, GC-5mC, and CC-5mC; Table S3), and 5hmC-containing DNA mixture (TC-5hmC, AC-5hmC, GC-5hmC, and CC-5hmC; Table S3). The DNA mixtures were separately treated with eA3A-v10 or wtA3A followed by LC–MS/MS analysis. The results showed that dC and 5mC signals were undetectable after eA3A-v10 treatment; however, the signal intensity of 5hmC was comparable to that with or without eA3A-v10 treatment (Figure 2A). In addition, other canonical nucleosides of dA, dG, and dT were not affected by eA3A-v10 treatment (Figure 2A). These results indicated that eA3A-v10 could efficiently deaminate C/5mC, but it showed no deamination activity to 5hmC, which is in line with the results of Sanger sequencing (Figure 1D). By contrast, wtA3A treatment led to the appreciable deamination of 5hmC in addition to the full deamination of C and 5mC (Figure S13).

We further treated these DNA mixtures with different concentrations of eA3A-v10 or wtA3A. The results revealed that the deamination percentages of C and 5mC were continuously increased and eventually reached to almost 100% with 1 μM of eA3A-v10; however, 5hmC showed no obvious deamination with the increased concentration of A3A-v10 (Figure 2B). However, it can be observed that wtA3A treatment also led to significant deamination of 5hmC in addition to C and 5mC (Figure 2B).

We next performed a quantitative evaluation of the deamination properties of eA3A-v10 and wtA3A to C, 5mC, and 5hmC by steady-state kinetic analysis. The results demonstrated that eA3A-v10 exhibited efficient deamination activity toward C (*k*_cat_/*K*_M_ = 6.27 μM^–1^ min^–1^) and 5mC (*k*_cat_/*K*_M_ = 3.90 μM^–1^ min^–1^) (Figure 2C). However, due to the extremely low activity of eA3A-v10 toward 5hmC, the kinetic parameters could not be obtained. The steady-state kinetics analysis of wtA3A revealed that wtA3A showed high deamination activity toward C (*k*_cat_/*K*_M_ = 90.82 μM^–1^ min^–1^) and 5mC (*k*_cat_/*K*_M_ = 22.45 μM^–1^ min^–1^) and also exhibited appreciable deamination activity toward 5hmC (*k*_cat_/*K*_M_ = 0.32 μM^–1^ min^–1^) (Figure S14). Collectively, the quantitative evaluation by steady-state kinetics analysis also demonstrated that eA3A-v10 exhibited distinctly differential deamination activity toward C, 5mC, and 5hmC.

### Development of SSD-seq

**Figure 3:**
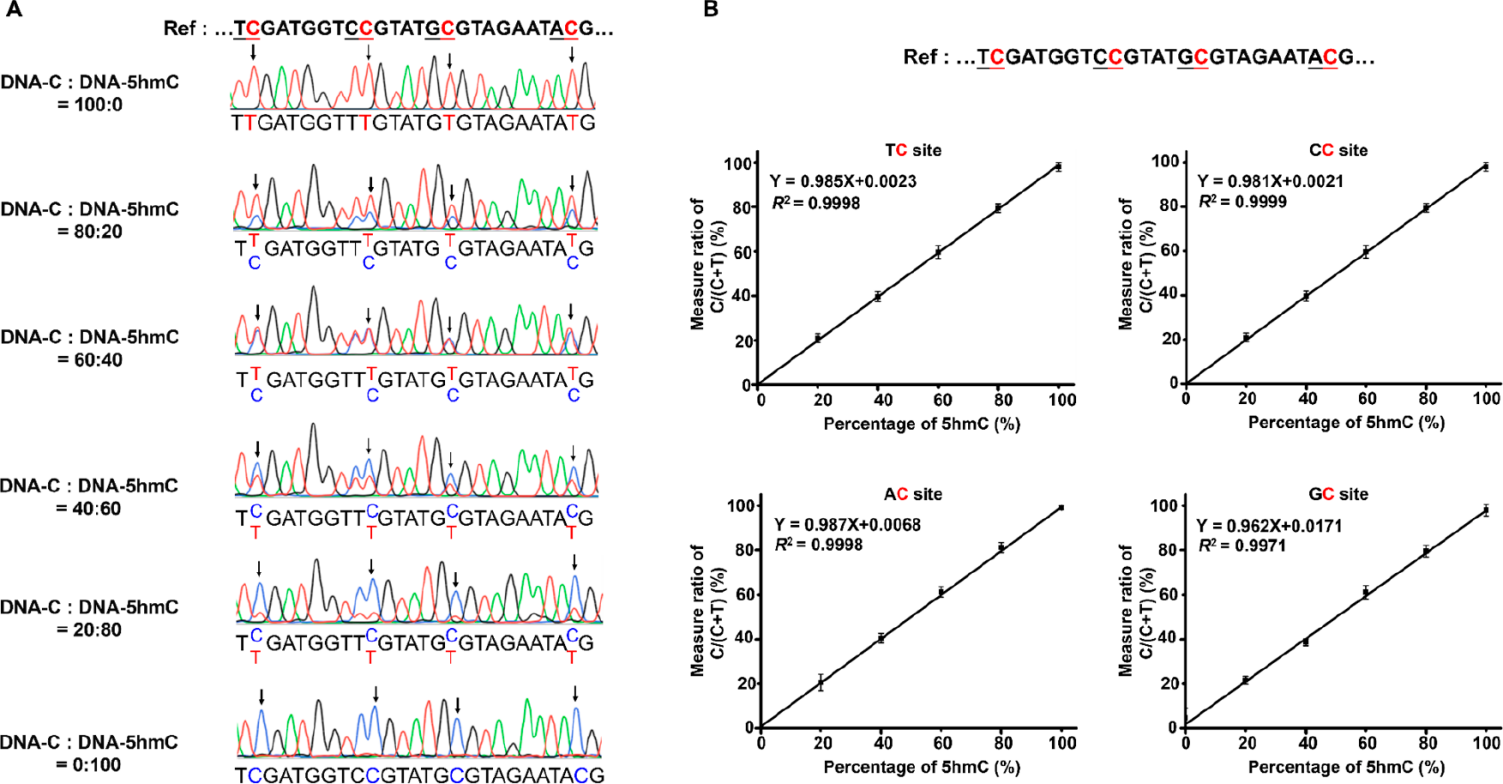
Quantitative evaluation of the level of 5hmC at different sequence contexts of TC, CC, GC, and AC in DNA by SSD-seq. (A) DNA-C and DNA-5hmC were mixed at different ratios with DNA-5hmC ranging from 0 to 100%. The mixtures were treated with eA3A-v10 followed by Sanger sequencing. (B) Linear regression of the measured ratios of C/(C + T) at individual sites with the theoretical percentages of 5hmC in the mixture of DNA-C and DNA-5hmC.

With the characterized eA3A-v10 that is capable of differentially deaminating C/5mC and 5hmC, we proposed the SSD-seq for the quantitative detection of 5hmC at single-base resolution in DNA. During the screening of eA3A-v10, the Sanger sequencing results clearly demonstrated that all of the C and 5mC in different sequence contexts of TC, AC, GC, and CC were read as T while all of the 5hmC in these sequence contexts were read as C. The preliminary results showed that the SSD-seq method is capable of single-base resolution detection of 5hmC. We then further evaluated the quantitative capability of the SSD-seq in measuring the stoichiometry of 5hmC at individual sites in DNA. In this respect, DNA-C and DNA-5hmC were mixed at different ratios, with DNA-5hmC ranging from 0 to 100%. The prepared mixtures were subjected to SSD-seq with Sanger sequencing (Figure 3A). The results showed that the measured ratio of C/(C + T) at individual sites increased linearly with the increased percentage of 5hmC in the mixture of DNA-C and DNA-5hmC (Figure 3A and 3B), suggesting that the SSD-seq method is capable of the quantitative measurement of 5hmC with different stoichiometries.

We also examined the detection capability of SSD-seq with a limited amount of DNA. In this respect, 100 ng, 1 ng, and 1 pg of DNA-C were separately treated by eA3A-v10 followed by PCR amplification. PCR products could be clearly detected even with 1 pg of DNA-C, DNA-5mC, and DNA-5hmC (Figure S15). Then, 1 pg of DNA-C, DNA-5mC, and DNA-5hmC was subjected to SSD-seq. The results showed that all of the C and 5mC sites were read as T while all of the 5hmC sites were read as C after the treatment of eA3A-10 (Figure S16). These results indicated that SSD-seq is capable of detecting 5hmC with a low amount of input DNA.

**Figure 4:**
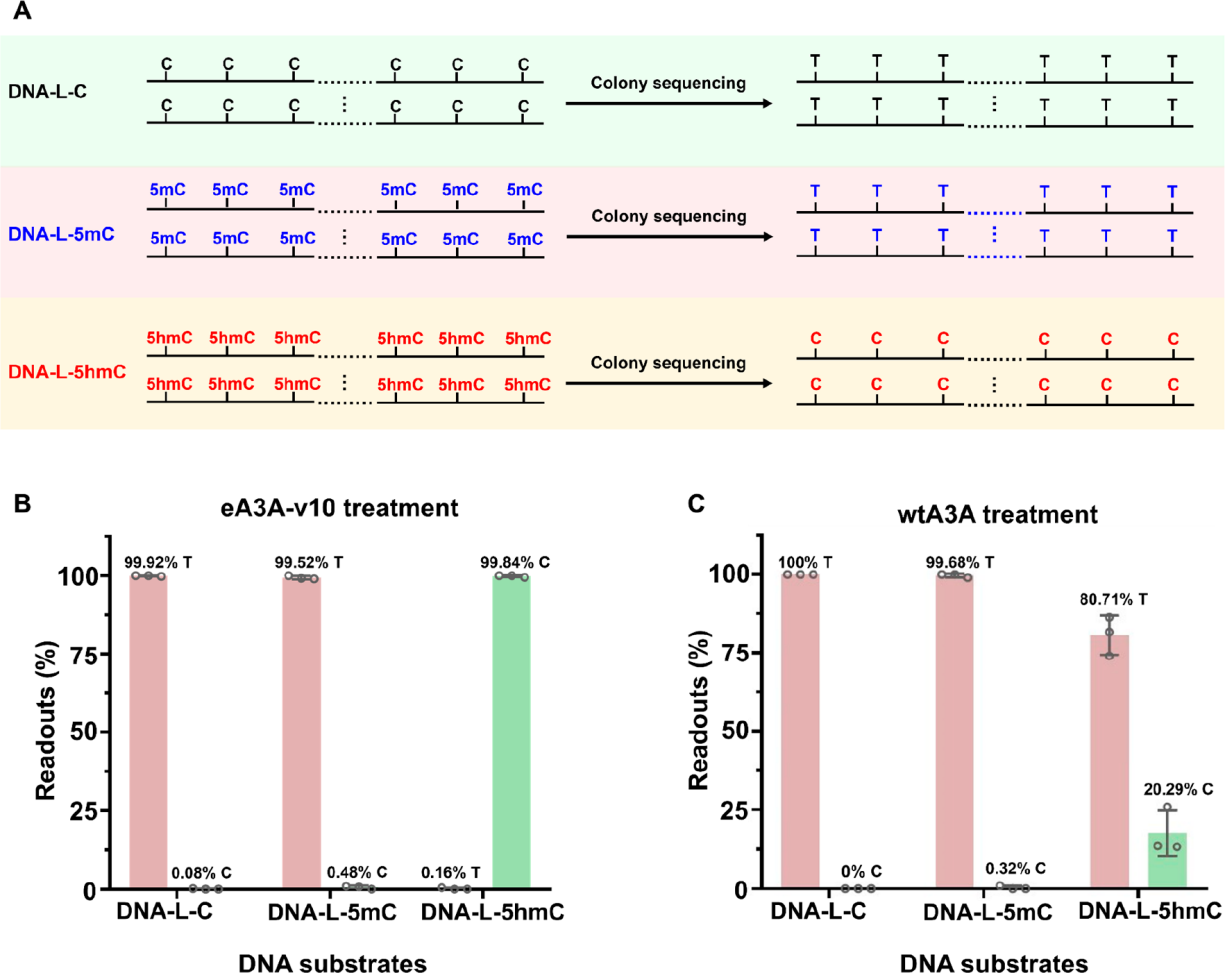
Quantitative evaluation of readouts of C, 5mC, and 5hmC in SSD-seq by colony sequencing. (A) Schematic illustration for the evaluation of the performance of SSD-seq by colony sequencing. DNA-L-C, DNA-L-5mC, and DNA-L-5hmC were treated with eA3A-v10 or wtA3A followed by colony sequencing. (B) Readouts of C, 5mC, and 5hmC after eA3A-v10 treatment with colony sequencing. (C) Readouts of C, 5mC, and 5hmC after wtA3A treatment with colony sequencing.

DNA substrates (DNA-C, DNA-5mC, and DNA-5hmC) used for the aforementioned evaluation carry only five C, 5mC, or 5hmC sites, which is a relatively simple system. Here we further employed three kinds of dsDNA (DNA-L-C, DNA-L-5mC, and DNA-L-5hmC; Table S4) that contain multiple numbers of C, 5mC, or 5hmC sites to evaluate the performance of SSD-seq. The DNA substrates were denatured and treated with eA3A-v10 or wtA3A followed by colony sequencing (Figure 4A). The results demonstrated that almost all of the C and 5mC were read as T by eA3A-v10 treatment, with the C-to-T and 5mC-to-T conversion rates being 99.92 and 99.52%, respectively (Figure 4B, Figures S17 and S18). As for 5hmC, the 5hmC-to-T conversion rate was only 0.16% (Figure 4B and Figure S19). Meanwhile, the C-to-T and 5mC-to-T conversion rates were 100.00 and 99.68% by wtA3A treatment (Figure 4C, Figures S20 and S21). However, the 5hmC-to-T conversion rate by wtA3A treatment was 80.71% (Figure 4C and Figure S22). The colony sequencing results demonstrated that eA3A-v10, but not wtA3A, could be used in SSD-seq for the direct detection of 5hmC at single-base resolution.

### Genome-Wide Mapping of 5hmC by SSD-seq

With the proposed SSD-seq, we carried out genome-wide mapping of 5hmC from human normal lung tissue. A 40 ng quantity of genomic DNA of lung tissue was spiked with 0.1% lambda bacteriophage DNA and then subjected to the SSD-seq analysis. Before eA3A-v10 treatment, 0.1% DNA-5mC and 0.1% DNA-L-5hmC were added as spike-ins to evaluate the deamination rates of 5mC and 5hmC. An average sequencing depth of ∼10× per strand was achieved (Table S5). The analysis of these spike-ins confirmed that the average C-to-T and 5mC-to-T conversion rates were 99.8 and 100%, respectively (Table S6). On the contrary, over 99.8% of 5hmC was intact and still read as C (Table S6).

**Figure 5:**
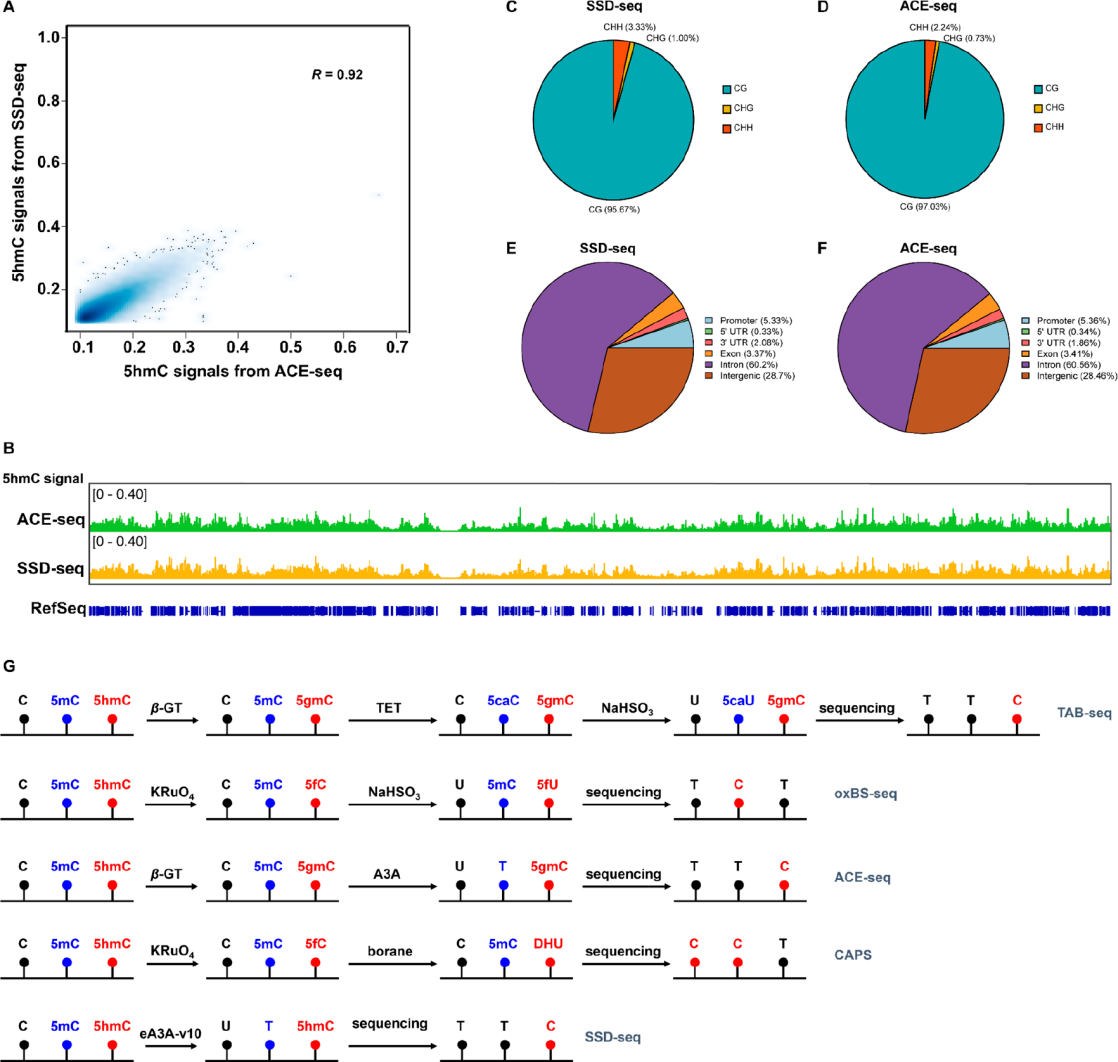
Genome-wide mapping of 5hmC by SSD-seq and comparison of analytical strategies of different 5hmC mapping methods. (A) Correlation 5hmC density plot in human normal lung tissue between SSD-seq and ACE-seq. (B) Snapshot of base-resolution 5hmC maps in chromosome 6 by SSD-seq and by ACE-seq. (C) Sequence context of statistically significant 5hmC sites from SSD-seq. (D) Sequence context of statistically significant 5hmC sites from ACE-seq. (E) Distribution of 5hmC sites in different gene regulatory elements and genomic features from SSD-seq. (F) Distribution of 5hmC sites in different gene regulatory elements and genomic features from ACE-seq. (G) Schematic illustration of analytical strategies of different 5hmC mapping methods.

For comparison, we also carried out the genome-wide mapping of 5hmC using previously developed ACE-seq.^34^ An average sequencing depth of ∼11× per strand was achieved (Table S5). The analysis of the spike-ins confirmed the C-to-T and 5mC-to-T in ACE-seq average conversion rates to be 99.90 and 99.95%, respectively (Table S6). On the contrary, all of the 5hmC sites were still read as C (Table S6). With a *q*-value cutoff of 0.01, 317,834 and 406,305 high-confidence 5hmC sites were called by SSD-seq and ACE-seq, respectively (Table S5). A comparison of the 5hmC sites from SSD-seq and ACE-seq showed a relatively good correlation (*r* = 0.92, Figure 5A). In addition, the distribution of 5hmC sites in different chromosomes from SSD-seq was similar to that from ACE-seq (Figure 5B and Figure S23).

Previous studies revealed that 5hmC in the mammalian genome occurs almost exclusively in CpG contexts. We found that the majority of 5hmC sites also occurred in CpG contexts by both SSD-seq (95.67%) and ACE-seq (97.03%) (Figure 5C and 5D). In addition, the distribution of 5hmC obtained by SSD-seq in different gene regulatory elements was also similar to that obtained by ACE-seq (Figure 5E and 5F). 5hmC sites called by both SSD-seq and ACE-seq were mainly enriched in the gene body (Figure 5E and 5F), which is in line with the function of 5hmC in the regulation of chromosome accessibility and gene expression. The genome-wide mapping of 5hmC by SSD-seq shared comparable results with ACE-seq, giving confidence to both techniques. We further conducted an analysis of the average 5hmC level around the transcriptional start sites (TSS). The results reveal a significant enrichment of 5hmC around the TSS (Figure S24). In line with previous studies, the presence of 5hmC in the vicinity of TSS implies its involvement in the modulation of transcriptional activity. We also conducted gene ontology (GO) enrichment analysis and pathway enrichment analysis (KEGG). The results reveal that 5hmC participates in a wide range of biological processes and pathways (Figures S25 and S26). The identification of enriched GO terms and pathways associated with 5hmC suggests that 5hmC may play crucial roles in regulating diverse biological processes and pathways, potentially influencing gene expression, cellular development, and disease progression.

Compared with previous methods used for mapping 5hmC, SSD-seq offers several notable advantages. Figure 5G summarizes the analytical strategies of various 5hmC mapping methods. First, SSD-seq enables the mapping of 5hmC at single-base resolution in DNA. In contrast, previous affinity enrichment-based methods do not provide information about the exact location of 5hmC at the single-base level. Second, SSD-seq simplifies the analytical process compared to methods such as ACE-seq and TAPSβ. The principle of SSD-seq is straightforward, and the experimental procedure is simple since it does not require the glycosylation of 5hmC, TET-mediated oxidation, or pyridine borane treatment. Third, SSD-seq differs from oxBS-seq and TAB-seq in that it utilizes a mild deamination reaction, eliminating the need for harsh chemical reactions such as bisulfite treatment during library construction. This gentle approach ensures that DNA is not susceptible to degradation, making SSD-seq suitable for 5hmC mapping analysis with limited amounts of input DNA, such as in single-cell 5hmC mapping studies. Fourth, SSD-seq demonstrates no sequence bias in 5hmC mapping. We observed no bias in the sequence context for eA3A-v10-mediated deamination of cytosines in DNA. Consequently, unlike methods reliant on restriction enzyme-based cleavage, which can map 5hmC only in specific sequence contexts,^41^ SSD-seq offers precise and comprehensive mapping of 5hmC in any sequence context. It is worth noting that in addition to 5hmC, 5fC and 5caC also are present in DNA. However, the levels of 5fC and 5caC are significantly lower than that of 5hmC, typically by 2 to 3 orders of magnitude.^42,43^ As a result, their impact on the mapping of 5hmC using SSD-seq is minimal and can be disregarded. Overall, SSD-seq overcomes several limitations present in previous 5hmC mapping methods, making it a cost-effective and versatile approach for the high-resolution analysis of 5hmC in DNA.

In summary, we successfully developed a method of SSD-seq, which utilizes the engineered eA3A-v10 protein, for the quantitative and genome-wide detection of 5hmC at single-base resolution. The map of 5hmC generated by SSD-seq in human lung tissue exhibited a strong correlation with the results obtained by using the ACE-seq method. Overall, SSD-seq is bisulfite-free and chemical labeling-free and does not require DNA glycosylation or chemical oxidation steps. This approach provides a valuable tool for the direct, cost-effective, and quantitative detection of 5hmC in DNA at single-base resolution. The SSD-seq method opens up the possibilities for using engineered DNA-modifying enzymes to develop novel methods for mapping DNA modifications, which expands the repertoire of available biotechnological approaches and holds promise for further advancements in the field of epigenetic research.

## Materials and Methods

### Materials and Reagents

Oligonucleotides that carry different cytosine modifications were purchased from Takara Biotechnology Co., Ltd. (Dalian, China). The detailed sequences of these oligonucleotides are listed in Table S3. 2′-Deoxycytidine (dC), thymidine (dT), 2′-deoxyguanosine (dG), 2′-deoxyadenosine (dA), 2′-deoxynucleoside 5′-triphosphates (dATP, dCTP, dGTP, and TTP), and phosphodiesterase I were purchased from Sigma-Aldrich (St. Louis, MO, USA). 5-Hydroxymethyl-2′-deoxycytidine-5′-triphosphate (5hmdCTP) and 5-methyl-2′-deoxycytidine-5′-triphosphate (5mdCTP) were purchased from TriLink BioTechnologies (San Diego, CA, USA). DNase I, S1 nuclease, and alkaline phosphatase (CIAP) were purchased from Takara Biotechnology Co. Ltd. (Dalian, China). EpiMark Hot Start *Taq* DNA polymerase, Q5U High-Fidelity DNA polymerase, and Q5 High-Fidelity DNA polymerase were purchased from New England Biolabs (Ipswich, MA, USA). The human normal lung tissue was collected from the Zhongnan Hospital of Wuhan University (Wuhan, China). All experiments were conducted in accordance with the guidelines and regulations of the Ethics Committee of Wuhan University. No unexpected or unusually high safety hazards were encountered.

### Preparation of DNA with C, 5mC, or 5hmC

Three 224-bp double-stranded DNA (dsDNA) substrates (DNA-C, DNA-5mC, and DNA-5hmC; Table S1) and three 367-bp dsDNA substrates (DNA-L-C, DNA-L-5mC, and DNA-L-5hmC; Table S4) were synthesized by PCR amplification. DNA-C, DNA-5mC, and DNA-5hmC were synthesized according to a previous report.^36^ As for the preparation of DNA-L-C, 0.5 ng of synthetic DNA (Takara) was used as the template for PCR amplification. PCR amplification was carried out in a 50 μL solution including 1 U of Q5U High-Fidelity DNA polymerase, 4 μL of dNTP (2.5 mM), 5 μL of 10× reaction buffer, 2 μL of 10 μM L-F primer, and 2 μL of 10 μM L-R primer (Table S7). DNA-L-5mC and DNA-L-5hmC were prepared by PCR amplification, with dCTP being replaced with 5mdCTP or 5hmdCTP, respectively. The PCR reaction consisted of 95 °C for 5 min, 30 cycles of 95 °C for 1 min, 60 °C for 1 min, and 68 °C for 1 min, followed by an elongation at 68 °C for 10 min. The PCR products were separated by agarose gel electrophoresis and recovered using a gel extraction kit (Omega Bio-Tek Inc., Norcross, GA, USA). As for the DNA-L-5mC and DNA-L-5hmC, all of the cytosines were replaced by 5mC or 5hmC (except for the cytosines in PCR primers).

### Expression and Purification of Wild-Type A3A and Engineered A3A Proteins

To obtain wild-type A3A (wtA3A, Gene ID: 200315) and engineered A3A proteins, the coding sequence of wtA3A protein or engineered A3A (eA3A) proteins was cloned into pET-41a(+) plasmid between SpeI and XhoI restriction enzyme digestion sites, and an additional human rhinovirus 3C protease (HRV 3C) digestion site was inserted between the glutathione S-transferase (GST) tag and wtA3A protein or eA3A protein (Figure S27). The plasmids for the expression of the recombinant wtA3A protein or eA3A proteins were transformed to the *Escherichia coli* (*E*. *coli*) BL21(DE3) *pLysS* strain. The sequences of the plasmid and the amino acid sequence of the eA3A-v10 protein are listed in Tables S8 and S9, respectively. The culturing of these transformed *E*. *coli* cells and the expression and purification of recombinant proteins were carried out according to our previous report,^36^ and the detailed procedures can be found in the Supporting Information. The purified proteins were stored at −80 °C in a solution containing 50 mM NaCl, 50 mM Tris-HCl (pH 7.5), 0.5 mM dithiothreitol, 0.01 mM EDTA, and 0.01% Tween-20. The purified proteins were determined by SDS-PAGE (Figure S28). The concentrations of purified proteins were quantified using a BCA protein assay kit (Beyotime, Shanghai, China).

### Evaluation of the Deamination Activities of eA3A Proteins by Sequencing

Three dsDNAs (DNA-C, DNA-5mC, and DNA-5hmC) were used as substrates to evaluate the deamination activities of wtA3A and eA3A proteins toward C, 5mC, and 5hmC by sequencing. Typically, 40 ng of dsDNA was first denatured to single-stranded (ssDNA) by heating to 95 °C for 10 min in a 20% dimethylsulfoxide (DMSO) (v/v) solution and chilling in ice water. Then, the deamination reaction was carried out at 37 °C for 2 h in a 20 μL solution of 20 mM 2-morpholinoethanesulfonate (MES) (pH 6.5), 2 μL of DMSO, 0.1% Triton X-100, and 20 μM of wtA3A or eA3A protein. The deamination reaction was terminated by heating to 95 °C for 10 min. Then, 5 ng of deaminase-treated DNA was used as the template for PCR amplification. PCR amplification was carried out in a 50 μL solution containing 10 μL of 5× reaction buffer, 1 U of EpiMark Hot Start *Taq* DNA polymerase, 0.2 mM dNTP, 0.4 μM A-F primer, and 0.4 μM A-R primer (Table S7). The PCR reaction included initial denaturation at 95 °C for 5 min, 30 cycles of 95 °C for 30 s, 55 °C for 30 s, and 68 °C for 1 min, and 10 min of additional elongation at 68 °C. The resulting PCR products were subjected to Sanger sequencing. In addition to Sanger sequencing, we also carried out colony sequencing to quantitatively evaluate the deamination efficiencies of C, 5mC, and 5hmC by wtA3A and eA3A proteins with DNA-L-C, DNA-L-5mC, and DNA-L-5hmC as substrates. The detailed procedures of colony sequencing can be found in the Supporting Information.

### Characterization of the Deamination Properties of eA3A Proteins by LC–MS/MS Analysis

A series of 24-mer C-containing DNA (GC-C, AC-C, CC-C, and TC-C), 5mC-containing DNA (GC-5mC, AC-5mC, CC-5mC, and TC-5mC), and 5hmC-containing DNA (GC-5hmC, AC-5hmC, CC-5hmC, and TC-5hmC) were utilized as substrates to characterize the deamination properties of eA3A proteins using LC–MS/MS analysis. Typically, 10 pmol of the C-containing DNA mixture, 5mC-containing DNA mixture, or 5hmC-containing DNA mixture was treated with wtA3A or eA3A proteins. The deamination reaction was carried out at 37 °C for 2 h in a 20 μL solution including 20 mM MES (pH 6.5), 2 μL of DMSO, and 0.1% Triton X-100. The reaction was terminated by heating to 95 °C for 10 min. The resulting DNA was enzymatically digested, followed by liquid chromatography–tandem mass spectrometry (LC–MS/MS) analysis according to the previously described protocol.^44^ The detailed procedures of the enzymatic digestion of DNA and LC–MS/MS analysis can be found in the Supporting Information.

### Steady-State Kinetic Study

Kinetic assays were performed with DNA mixtures containing different cytosine modifications (GC-C, AC-C, CC-C, and TC-C; GC-5mC, AC-5mC, CC-5mC, and TC-5mC; and GC-5hmC, AC-5hmC, CC-5hmC, and TC-5hmC). As for the C-containing DNA mixture and 5mC-containing DNA mixture, different concentrations of substrates (from 100 nM to 2.5 μM) were treated with 10 nM wtA3A or 40 nM eA3A-v10 at 37 °C for 5 min in 20 mM MES (pH 6.5) buffer. As for the 5hmC-containing mixture, different concentrations of substrates (from 25 nM to 1.25 μM) were treated with 1 μM wtA3A or 10 μM eA3A-v10 at 37 °C for 5 min in 20 mM MES (pH 6.5) buffer. The reaction was terminated by heating to 95 °C for 10 min, and the resulting DNA was enzymatically digested, followed by LC–MS/MS analysis.

The deamination rates of C, 5mC, and 5hmC by wtA3A or eA3A were calculated from the ratio of the deaminated product (*I*_D_) over the undeaminated product (*I*_U_) plus the deaminated product (*I*_D_) as follows: deamination rate*([E])*t = *I*_D_/(*I*_U_ + *I*_D_), where *t* represents the reaction time and [E] represents the concentration of deaminase. The apparent *K*_M_ and *k*_cat_ values were obtained from linear regression analysis of the Michaelis–Menten equation [deamination rate = (*k*_cat_)([S])/(*K*_M_ + [S])] using the data points at different DNA concentrations in three independent experiments according to a previously described method.^45,46^ The [S] in the Michaelis–Menten equation represents the concentration of DNA substrates. The enzymatic efficiency (*k*_cat_/*K*_m_) was used to describe the selectivity of deaminases for deaminating C, 5mC, or 5hmC.

### Sequencing Library Construction for SSD-seq

Genomic DNA of human normal lung tissue was extracted using a tissue DNA kit (Omega Bio-Tek Inc., Norcross, GA, USA) according to the manufacturer’s recommended procedure. The unmodified genomic DNA of the lambda bacteriophage (Sangon Biotech, Shanghai, China) was added to the genomic DNA of human normal lung tissue as a spike-in control (0.1% of spike-in DNA was added). The mixture was sheared to an average size of 250–400 bp by using a JY92-II N ultrasonic homogenizer (Scientz Biotechnology Co., Ltd., China). The resulting fragmented DNA was end-repaired and adenylated using a Hieff NGS Ultima Endprep Mix Kit (Yeasen Biotechnology Co., Ltd., Shanghai). Then, an SSD-adaptor (Table S7) was ligated to both ends of repaired DNA using a Hieff NGS Ultima DNA Ligation Module Kit (Yeasen), and the resulting DNA was purified using 0.8× KAPA Pure beads (Roche). To the resulting mixture were added DNA-L-5mC and DNA-5hmC as spike-ins to evaluate the deamination rates of 5mC and 5hmC (0.1% DNA-L-5mC and 0.1% DNA-5hmC were added).

The DNA mixture was denatured, followed by deamination using eA3A-v10. The deaminated DNA was amplificated by PCR with five cycles using pre-P5 primer, pre-P7 primer (Table S7), and Q5U Hot Start High-Fidelity DNA polymerase (New England Biolabs). After purification using 0.8× KAPA Pure beads, DNA products were then amplificated by PCR with 10 cycles using P5-index primer, P7-index primer (Table S7), and Q5 Hot Start High-Fidelity DNA polymerase (New England Biolabs). The PCR products were purified with 0.8× KAPA Pure beads and examined using 1.5% agarose gel electrophoresis. Library quality was assessed on an Agilent Bioanalyzer 2100 system. The library was then sequenced on an Illumina NovaSeq 6000 platform (Novogene Co., Ltd., Nanjing, China). The schematic diagram of library preparation is shown in Figure S29. In addition, we also carried out the genome-wide mapping of 5hmC with a previously developed ACE-seq method.^34^ The sequencing library construction for ACE-seq can be found in Figure S30 in the Supporting Information. The SSD-seq and ACE-seq data have been deposited into the NCBI Gene Expression Omnibus (GEO) under accession number GSE236353.

### Data Analysis

Sequencing reads were processed according to previous reports.^34,47^ Briefly, the data quality was examined with FastQC (v0.11.8) software (https://www.bioinformatics.babraham.ac.uk). Low-quality bases and adaptor sequences were removed from the raw reads using Trimmomatic (version 0.39).^48^ The trimmed reads were mapped against the reference genomes (hg19) with Bismark (v0.23.0).^49^ PCR duplicates and overlapping read pairs were removed and clipped using Bismark and BamUtil (version 1.0.14),^50^ respectively, and 5hmC raw signals were calculated as the percentage of C at each site.

For each original cytosine site, the number of C reads from SSD-seq and ACE-seq was counted as 5hmC (denoted N_C_) and the number of T reads was counted as 5mC or unmodified cytosine (denoted N_T_). The sequencing depth and coverage of samples were calculated using Bismark and samtools (v1.9) software.^51^ High-confidence 5hmC sites (depth ≥ 5) were called using a binomial distribution with a *q*-value cutoff of 0.01 according to the previous report.^52^ For the comparison between SSD-seq and ACE-seq, the high-confidence 5hmC signals were calculated within tiled 10 kb genomic bins according to the previous study.^34^ Pearson correlation coefficients were calculated using the R function cor. The Integrative Genomics Viewer (IGV, v2.9.2)^53^ was used to visualize signals from SSD-seq and ACE-seq with hg19 Refseq transcript annotation as reference. 5hmC sites are indicated by upward ticks, with the height of each tick representing the fraction of 5hmC at the site ranging from 0 to 0.4. The CpGs were annotated using the ChIPseeker package based on the distance to the closest transcriptional start site.^54^

## Data Availability

The expression plasmids for wtA3A and eA3A-v10 are freely available upon request. The SSD-seq and ACE-seq data have been deposited in the NCBI Gene Expression Omnibus (GEO) under accession number GSE236353.

## Supporting Information Available

Expression and purification of wild-type A3A and engineered A3A proteins; enzymatic digestion of DNA; LC–MS/MS analysis; sequencing library construction for ACE-seq; evaluation of the deamination activities of wtA3A and eA3A proteins by colony sequencing; and Tables S1–S9 and Figures S1–S30 (PDF)
