# Supplementary material for: Whole-Genome Sequencing of 5-Hydroxymethylcytosine at Base Resolution by Bisulfite-Free Single-Step Deamination with Engineered Cytosine Deaminase
Source: ACS Cent Sci. 2023 Nov 30;9(12):2315–25. doi: 10.1021/acscentsci.3c01131 (PMC10755730; doi:10.1021/acscentsci.3c01131)
Supplement: Supplementary file 1 — oc3c01131_si_001.pdf [file oc3c01131_si_001.pdf]

Supporting Information

for

Whole-genome sequencing of 5-hydroxymethylcytosine at base resolution by bisulfite-free single-step deamination with engineered cytosine deaminase

Neng-Bin Xie,[1,2,†] Min Wang,[3,†] Wei Chen,[4,†] Tong-Tong Ji,[3] Xia Guo,[3] Fang-Yin Gang,[1] Ya-Feng Wang,[1] Yu-Qi Feng,[1] Yuan Liang,[5,6,*] Weimin Ci,[5,6,*] Bi-Feng Yuan,[1,2,3,*]

[1] Department of Occupational and Environmental Health, School of Public Health, Wuhan University, Wuhan, 430071, China.

[2] Research Center of Public Health, Renmin Hospital of Wuhan University, Wuhan University, Wuhan 430060, China.

[3] College of Chemistry and Molecular Sciences, Wuhan University, Wuhan 430072, China

[4] Department of Laboratory Medicine, Zhongnan Hospital of Wuhan University, Wuhan University, Wuhan 430071, China.

[5] Key Laboratory of Genomics and Precision Medicine, and China National Center for Bioinformation, Beijing Institute of Genomics, Chinese Academy of Sciences, Beijing 100101, China.

[6] University of Chinese Academy of Sciences, Beijing 100049, China.

[†] These authors contributed equally to this work.

* Corresponding author:

Bi-Feng Yuan. E-mail: bfyuan@whu.edu.cn

Weimin Ci. E-mail: ciwm@big.ac.cn

Yuan Liang. E-mail: liangyuan@big.ac.cn

Table of Contents

Page S4-S7	Expression and purification of wild-type A3A and engineered A3A proteins; Enzymatic digestion of DNA; LC-MS/MS analysis; Sequencing library construction for ACE-seq; Evaluation of the deamination activities of wtA3A and eA3A proteins by colony sequencing.
Page S8	Table S1. Sequences of DNA-C, DNA-5mC and DNA-5hmC.
Page S9	Table S2. The deamination characteristics of eA3A proteins.
Page S10	Table S3. Sequences of oligonucleotides.
Page S11	Table S4. Sequences of DNA-L-C, DNA-L-5mC, and DNA-L-5hmC.
Page S12	Table S5. Sequencing data by SSD-seq and ACE-seq.
Page S13	Table S6. Conversion rates of C, 5mC and 5hmC in the spiked-in DNA in SSD-seq and ACE-seq by colony sequencing.
Page S14	Table S7. Sequences of PCR primers and SSD-adaptor.
Page S15	Table S8. Sequence information of the plasmid used for expressing the eA3A-v10 protein.
Page S16	Table S9. Sequence information of the amino acid composition of the eA3A-v10 protein.
Page S17	Figure S1. Characterization of the deaminase selectivity of wtA3A toward C, 5mC and 5hmC in different sequence context by Sanger sequencing.
Page S18	Figure S2. Principle of the engineered deaminase-mediated sequencing (EDM-seq).
Page S19	Figure S3. Characterization of the deaminase selectivity of eA3A-v1 toward C, 5mC and 5hmC in different sequence context by Sanger sequencing.
Page S20	Figure S4. Characterization of the deaminase selectivity of eA3A-v2 toward C, 5mC and 5hmC in different sequence context by Sanger sequencing.
Page S21	Figure S5. Characterization of the deaminase selectivity of eA3A-v3 toward C, 5mC and 5hmC in different sequence context by Sanger sequencing.
Page S22	Figure S6. Characterization of the deaminase selectivity of eA3A-v4 toward C, 5mC and 5hmC in different sequence context by Sanger sequencing.
Page S23	Figure S7. Characterization of the deaminase selectivity of eA3A-v5 toward C, 5mC and 5hmC in different sequence context by Sanger sequencing.
Page S24	Figure S8. Characterization of the deaminase selectivity of eA3A-v6 toward C, 5mC and 5hmC in different sequence context by Sanger sequencing.
Page S25	Figure S9. Characterization of the deaminase selectivity of eA3A-v7 toward C, 5mC and 5hmC in different sequence context by Sanger sequencing.
Page S26	Figure S10. Characterization of the deaminase selectivity of eA3A-v8 toward C, 5mC and 5hmC in different sequence context by Sanger sequencing.
Page S27	Figure S11. Characterization of the deaminase selectivity of eA3A-v9 toward

	C, 5mC and 5hmC in different sequence context by Sanger sequencing.
Page S28	Figure S12. The amino acid composition of wtA3A and eA3A-v10 proteins.
Page S29	Figure S13. Extracted-ion chromatograms of dC, 5mC, 5hmC, dA, dG and dT from wtA3A-treated and untreated DNA by LC-MS/MS analysis.
Page S30	Figure S14. Quantitative evaluation of the deamination activity of wtA3A toward C, 5mC and 5hmC using steady-state kinetics analysis.
Page S31	Figure S15. Agarose gel electrophoresis analysis of the PCR products from different amounts of DNA treated with eA3A-v10.
Page S32	Figure S16. Evaluation of the detection capability of the SSD-seq with small amount of DNA. 1 pg of DNA-C, DNA-5mC and DNA-5hmC were subjected to SSD-seq analysis.
Page S33	Figure S17. Evaluation of the deaminase activity of eA3A-v10 toward C by colony sequencing.
Page S34	Figure S18. Evaluation of the deaminase activity of eA3A-v10 toward 5mC by colony sequencing.
Page S35	Figure S19. Evaluation of the deaminase activity of eA3A-v10 toward 5hmC by colony sequencing.
Page S36	Figure S20. Evaluation of the deaminase activity of wtA3A toward C by colony sequencing.
Page S37	Figure S21. Evaluation of the deaminase activity of wtA3A toward 5mC by colony sequencing.
Page S38	Figure S22. Evaluation of the deaminase activity of wtA3A toward 5hmC by colony sequencing.
Page S39	Figure S23. The distribution of 5hmC sites in different chromosomes obtained from SSD-seq and ACE-seq.
Page S40	Figure S24. The average 5hmC level around the transcriptional start sites (TSS).
Page S41	Figure S25. The Gene Ontology and pathway enrichment analysis of 5hmC sites in promotor region.
Page S42	Figure S26. The Gene Ontology and pathway enrichment analysis of 5hmC sites in gene body.
Page S43	Figure S27. The schematic illustration of plasmid for the expression of wtA3A or eA3A proteins.
Page S44	Figure S28. SDS-PAGE analysis of the purified wtA3A and eA3A-v10.
Page S45	Figure S29. The schematic diagram of library preparation in SSD-seq.
Page S46	Figure S30. The schematic diagram of library preparation in ACE-seq.
Page S47	References

Methods

Expression and purification of wild-type A3A and engineered A3A proteins

The coding sequence of wtA3A protein or engineered A3A (eA3A) proteins was cloned into pET-41a(+) plasmid between SpeI and XhoI restriction enzyme digestion sites and an additional human rhinovirus 3C protease (HRV 3C) digestion site was inserted between the glutathione S-transferase (GST) tag and wtA3A protein or eA3A protein (Figure S27). The plasmids for the expression of recombinant wtA3A protein or eA3A proteins were transformed into *Escherichia coli* (*E. coli*) BL21(DE3) *pLysS* strain. Transformed *E. coli* cells were cultured using LB medium (tryptone 10 g L^{-1}, yeast extract 5 g L^{-1}, and NaCl 10 g L^{-1}) supplemented with kanamycin (10 μg mL^{-1}) and chloramphenicol (10 μg mL^{-1}) at 37ºC under shaking at 180 rpm. Protein expression was started by the addition of 0.5 mM isopropyl-β-D-thiogalactoside (IPTG, Sangon) when the OD600nm of *E. coli* cell suspension reached 0.4-0.6. The expression of recombinant proteins was induced 20 h at 25ºC under shaking at 180 rpm. The *E. coli* cells were pelleted by centrifugation at 10000 g for 5 min and cell pellets were lysed by sonication in PBS buffer with 1 mM dithiothreitol and 50 μg mL^{-1} phenylmethylsulfonyl fluoride (PMSF). The supernatant was obtained by centrifugation at 12000 g for 30 min and filtered with a 0.22 μm membrane. The obtained supernatant was then incubated with Glutathione Sepharose 4B beads (Sangon, Shanghai, China) according to the manufacturer's recommended procedure. Recombinant proteins were concentrated on the beads. After the digestion with HRV 3C protease (Sangon, Shanghai, China), wtA3A or eA3A proteins were released from the beads and then further purified with a size-exclusion column (Millipore, Darmstadt, Germany) and equilibrated with a storage solution containing 50 mM Tris–HCl (pH 7.5), 50 mM NaCl, 0.01 mM EDTA, 0.5 mM dithiothreitol, and 0.01% Tween-20.

Enzymatic digestion of DNA

Deaminase-treated and untreated DNA were digested in a 50-μL solution with 4 U of DNase I, 30 U of alkaline phosphatase, 360 U of S1 nuclease, 0.002 U of venom phosphodiesterase I, and 50 μL of enzymatic digestion buffer (500 mM Tris-HCl, 100 mM NaCl, 10 mM MgCl2, 10 mM ZnSO4, pH 7.0). The mixture was incubated at 37℃ for 4 h

and 95℃ for 30 min. After adding 150 μL of H_2O, the resulting solution was extracted with chloroform three times. The resulting aqueous layer was collected and lyophilized to dryness and then reconstituted in 20 μL of water.

LC-MS/MS analysis

The analysis of nucleosides was performed on an LC-MS/MS system consisting of a Shimadzu 8045 mass spectrometer (Kyoto, Japan) with an electrospray ionization source (Turbo Ionspray) coupled with a Shimadzu LC-30AD UPLC system. Nucleosides were separated on a Shim-C18 column (Shim-pack GIST C18, 2.1×100 mm, 2.0 μm, Shimadzu, Japan) at 40°C with a flow rate of 0.3 mL/min. 0.05% formic acid (v/v, solvent A) and methanol (solvent B) were employed as mobile phases. A gradient of 0-1.5 min, 5% B, 1.5-3 min, 5%-40% B, 3-5 min, 40% B, 5-12 min, 40%-5% B, 12-20 min, 5% B were used for the separation. The MS detection was performed in positive ion mode with the interface temperature was set at 300°C and the collision-induced dissociation gas was optimized to 230 kPa. The voltage of the electrospray capillary was set as 4.0 kV. Nucleosides were monitored under multiple reaction monitoring (MRM) mode. Mass transitions (precursor ions → product ions) of dA (252.1 → 136.1), dG (268.1 → 152.1), dC (228.1 → 112.1), dT (243.1 → 127.1), 5mC (242.1 → 126.1) and 5hmC (258.1 →142.1) were utilized to determine the nucleosides.

Sequencing library construction for ACE-seq

Genomic DNA of human normal lung tissue was extracted using Tissue DNA kit (Omega Bio-Tek Inc., Norcross, GA, USA) according to the manufacturer's recommended procedure. The unmodified genomic DNA of lambda bacteriophage (Sangon Biotech, Shanghai, China) was added to the genomic DNA of human normal lung tissue as spike-in control (0.1% of spike-in DNA was added). The mixture was sheared to an average size of 250-400 bp using a JY92-II N Ultrasonic Homogenizer (Scientz Biotechnology Co., Ltd, China). The resulting fragmented DNA was end-repaired and adenylated using a Hieff NGS Ultima Endprep Mix kit (Yeasen Biotechnology Co., Ltd, Shanghai). Then, SSD-adaptor (Table S7) was ligated to both ends of repaired DNA using a Hieff NGS Ultima DNA Ligation Module kit (Yeasen) and the resulting DNA was purified using 0.8× KAPA Pure

beads (Roche). To the resulting mixture, DNA-L-5mC and DNA-5hmC were added as spike-in (0.1% of DNA-L-5mC and 0.1% DNA-5hmC were added). Then, the glycosylation of 5hmC was carried out using *β*-GT (New England Biolabs) at 37ºC for 2 h according to previous study.[1] The resulting DNA was purified using 0.8× KAPA Pure beads (Roche).

The DNA mixture was denatured followed by deamination using wtA3A. The deaminated DNA was amplificated by PCR with 5 cycles using pre-P5 primer, pre-P7 primer (Table S7) and Q5U Hot Start High-Fidelity DNA polymerase (New England Biolabs). After purification using 0.8× KAPA Pure beads, DNA products were then amplificated by PCR with 10 cycles using P5-index primer, P7-index primer (Table S7) and Q5 Hot Start High-Fidelity DNA polymerase (New England Biolabs). The PCR products were purified with 0.8× KAPA Pure beads and examined using 1.5% agarose gel electrophoresis. Library quality was assessed on the Agilent Bioanalyzer 2100 system. The library was then sequenced on an Illumina NovaSeq 6000 platform (Novogene Co., Ltd, Nanjing, China). The schematic diagram of library preparation of SSD-seq is shown in Figure S29.

Evaluation of the deamination activities of wtA3A and eA3A proteins by colony sequencing

The deaminase activities of wtA3A and eA3A proteins towards C, 5mC and 5hmC were evaluated using colony sequencing. Three kinds of dsDNA (DNA-L-C, DNA-L-5mC, and DNA-L-5hmC) were used as substrates. Briefly, 40 ng of dsDNA was denatured to single-stranded (ssDNA) by heating at 95°C for 10 min in a 20% dimethylsulfoxide (DMSO) (v/v) solution and chilling in ice water. Then, the deamination reaction was carried out at 37°C for 2 h in a 20-μL solution of 20 mM 2-morpholinoethanesulfonate (MES) (pH 6.5), 2 μL of DMSO, 0.1% Triton X-100, and 20 μM of wtA3A or eA3A proteins. The deamination reaction was terminated by heating at 95°C for 10 min. 5 ng of deaminase-treated DNA was taken as the template for PCR amplification using EpiMark Hot Start *Taq* DNA polymerase. PCR amplification was carried out in a 50-μL solution containing 10 μL of 5× reaction buffer, 1 U of EpiMark Hot Start *Taq* DNA polymerase, 0.2 mM dNTP, 0.4 μM L-A-F primer and 0.4 μM L-R primer (Table S7). PCR reaction included initial denaturation at 95°C for 5 min, 30 cycles of 95°C for 30 s, 55°C for 30 s and 68°C for 1 min, followed by 10 min of additional

elongation at 68°C. The resulting PCR products were subjected to colony sequencing. Colony sequencing was carried out according to our previous report.[2] Ten clones of each sample were randomly picked and subjected to sequencing.

Table S1. Sequences of DNA-C, DNA-5mC and DNA-5hmC.

Name	Sequence (5' to 3')
DNA-C	GAGTGACGCTGAGCTTGACGTCGCGCGATGAGAGGTGA TTATGAGTATGTATAGTGTTAGGAAGAGTGTAGTAATAG GATGAAGATGATTATATGATCGATGGTCCGTATGCGTAG AATACGTTGTTGTAGTGATTATAATGGAGTGAGAATGTA GATGAGTGGAGTAGGTAGTAAGATGTAGTGGTGAAGAG AGTAATTGTTAGTGGAATGTTGGAGAGGAT
DNA-5mC	GAGTGACGCTGAGCTTGACGTCGCGCGATGAGAGGTGA TTATGAGTATGTATAGTGTTAGGAAGAGTGTAGTAATAG GATGAAGATGATTATATGAT5mCGATGGT5mC5mCGTAT G5mCGTAGAATA5mCGTTGTTGTAGTGATTATAATGGAG TGAGAATGTAGATGAGTGGAGTAGGTAGTAAGATGTAG TGGTGAAGAGAGTAATTGTTAGTGGAATGTTGGAGAGG AT
DNA-5hmC	GAGTGACGCTGAGCTTGACGTCGCGCGATGAGAGGTGA TTATGAGTATGTATAGTGTTAGGAAGAGTGTAGTAATAG GATGAAGATGATTATATGAT5hmCGATGGT5hmC5hmCGT ATG5hmCGTAGAATA5hmCGTTGTTGTAGTGATTATAATG GAGTGAGAATGTAGATGAGTGGAGTAGGTAGTAAGATG TAGTGGTGAAGAGAGTAATTGTTAGTGGAATGTTGGAG AGGAT

Table S2. The deamination characteristics of eA3A proteins.

Name of eA3A protein	Deamination activity to C	Deamination activity to 5mC	Deamination activity to 5hmC
eA3A-v1	Full	Full	Partial
eA3A-v2	Full	Partial	None
eA3A-v3	Full	Partial	None
eA3A-v4	Full	Full	Partial
eA3A-v5	Full	Full	Partial
eA3A-v6	Full	Full	Partial
eA3A-v7	Full	Full	Partial
eA3A-v8	Full	Full	Partial
eA3A-v9	Full	Partial	None
eA3A-v10	Full	Full	None

Table S3. Sequences of oligonucleotides.

Name	Sequence (5' to 3')
GC-C	GTATGATGCGAATGAGATGTATTG
AC-C	GTATGATACGAATGAGATGTATTG
TC-C	GTATGATTCGAATGAGATGTATTG
CC-C	GTATGATCCGAATGAGATGTATTG
GC-5mC	GTATGATG5mCGAATGAGATGTATTG
AC-5mC	GTATGATA5mCGAATGAGATGTATTG
TC-5mC	GTATGATT5mCGAATGAGATGTATTG
CC-5mC	GTATGATC5mCGAATGAGATGTATTG
GC-5hmC	GTATGATG5hmCGAATGAGATGTATTG
AC-5hmC	GTATGATA5hmCGAATGAGATGTATTG
TC-5hmC	GTATGATT5hmCGAATGAGATGTATTG
CC-5hmC	GTATGATC5hmCGAATGAGATGTATTG

Note: the "C" represented 5'-aza-2'-deoxycytidine.

Table S4. Sequences of DNA-L-C, DNA-L-5mC, and DNA-L-5hmC.

Name	Sequence (5’ to 3’)
DNA-L-C	AGTGACGCTGAGCTTGACGTCGCGCGATGAGAGGTGATTATGAGTACGTATAGTGTTAGGATAGAGTTCGTGTAGTAATAGGATGATAGTATGATTATATGAATGGTGAGTATGTGTAGAATCAGAGAGTAATTGTTAGTGGAATGTTGGGGATCCTCTAGAGTCGACCTGCAGGCATGCAAGCTTGGCGTAATCATGGTCATAGCTGTTTCCTGTGTGAAATTGTTATCCGCTCACAATTCCACACAACATACGAGCCGGAAGCATAAAGTGTAAAGCCTGGGGTGCCTAATGAGTGAGCTAACTCACATTAATTGCGTTGTGTTTAATGTTTGTTTTTAGTTGGGAAATTTGTTGTGAGAGAGT
DNA-L-5mC	AGTGACGCTGAGCTTGACGTCGCGCGATGAGAGGTGATTATGAGTA5mCGTATAGTGTTAGGATAGAGTT5mCGTGTAGTAATAGGATGATAGTATGATTATATGAATGGTGAGTATGTGTAGAAT5mCAGAGAGTAATTGTTAGTGGAATGTTGGGGAT5mC5mCT5mCTAGAGT5mCGA5mC5mCTG5mCAGG5mCATG5mCAAG5mCTTGG5mCGTAAT5mCATGGT5mCATAG5mCTGTTT5mC5mCTGTGTGAAATTGTTAT5mC5mCG5mCT5mCA5mCAATT5mC5mCA5mCA5mCAA5mCATA5mCGAG5mC5mCGGAAG5mCATAAAGTGTAAAG5mC5mCTGGGGTG5mC5mCTAATGAGTGAG5mCTAA5mCT5mCA5mCATTAATTG5mCGTTGTGTTTAATGTTTGTTTTTAGTTGGGAAATTTGTTGTGAGAGAGT
DNA-L-5hmC	AGTGACGCTGAGCTTGACGTCGCGCGATGAGAGGTGATTATGAGTA5hmCGTATAGTGTTAGGATAGAGTT5hmCGTGTAGTAATAGGATGATAGTATGATTATATGAATGGTGAGTATGTGTAGAAT5hmCAGAGAGTAATTGTTAGTGGAATGTTGGGGAT5hmC5hmCT5hmCTAGAGT5hmCGA5hmC5hmCTG5hmCAGG5hmCATG5hmCAAG5hmCTTGG5hmCGTAAT5hmCATGGT5hmCATAG5hmCTGTTT5hmC5hmCTGTGTGAAATTGTTAT5hmC5hmCG5hmCT5hmCA5hmCAATT5hmC5hmCA5hmCA5hmCAA5hmCATA5hmCGAG5hmC5hmCGGAAG5hmCATAAAGTGTAAAG5hmC5hmCTGGGGTG5hmC5hmCTAATGAGTGAG5hmCTAA5hmCT5hmCA5hmCATTAATTG5hmCGTTGTGTTTAATGTTTGTTTTTAGTTGGGAAATTTGTTGTGAGAGAGT

Table S5. Sequencing data by SSD-seq and ACE-seq.

Methods	Number of detected 5hmC sites	Coverage	Depth of coverage
SSD-seq	317,834	89.4%	10.332
ACE-seq	406,305	89.5%	11.390

Table S6. Conversion rates of C, 5mC and 5hmC in the spiked-in DNA in SSD-seq and ACE-seq by colony sequencing.

Methods	C-to-T conversion rate	5mC-to-T conversion rate	Non-conversion rate of 5hmC	Non-conversion rate of 5hmC
SSD-seq	99.80%	100.00%	99.80%	-
ACE-seq	99.90%	99.95%	-	100.00%

Table S7. Sequences of PCR primers and SSD-adaptor.

Name	Sequence
L-F	5'-AGTGACGCTGAGCTTGACGTCGCGC-3'
L-R	5'-ACTCTCTCACAACAAATTTCCCAACTAAAAAAC-3'
L-A-F	5'-AGTGATGCTGAGCTTGATGTTGTGT-3'
A-F	5'-GAGTGATGTTGAGTTTGATGTTGTGT-3'
A-R	5'-CTCCAACATTCCACTAACAATTACTCTCT-3'
SSD-adaptor	5'-TGAGAGAGAGGAGAATATAAATGTCATCGAT-3'<br>3'-AGGAGAGGAGGATTATATTTACAGTAGCTp-5'
pre-P5	5'-CACTCTTTCCCTACACGACGCTCTTCCGATCTGAGAGAGAGGAGAATATAAATGTTATTGAT-3'
pre-P7	5'-GACTGGAGTTCAGACGTGTGCTCTTCCGATCTCCTCTCCTCCTAATATAAATATCATCA-3'
P5	5'-AATGATACGGCGACCACCGAGATCTACACTATAGCCTACACTCTTTCCCTACACGACGCTCTTCCGATCT-3'
P7	5'-CAAGCAGAAGACGGCATACGAGATCGAGTAATGTGACTGGAGTTCAGACGTGTGCTCTTCCGATCT-3'

Table S8. Sequence information of the plasmid used for expressing the eA3A-v10 protein.

Name	Sequence (5’ to 3’)
eA3A-v10 expression plasmid	cccccggccacggggcctgccaccatacccacgccgaaacaagcgctcatgagcccgaa<br>gtggcgagcccgatcttccccatcggtgatgtcggcgatataggcgccagcaaccgcacct<br>gtggcgccggtgatgccggccacgatgcgtccggcgtagaggatcgagatcgatctcgatc<br>ccgcgaaattaatacgactcactataggggaattgtgagcggataacaattcccctctagaaat<br>aattttgtttaactttaagaaggagatatacatatgtcccctatactaggttattggaaaattaagg<br>gccttgtgcaacccactcgacttcttttggaatatcttgaagaaaaatatgaagagcatttgtatg<br>agcgcgatgaaggtgataaatggcgaaacaaaaagtttgaattgggtttggagtttcccaatct<br>tccttattatattgatggtgatgttaaattaacacagtctatggccatcatacgttatatagctgaca<br>agcacaacatgttgggtggttgtccaaaagagcgtgcagagatttcaatgcttgaaggagcg<br>gttttggatattagatacggtgtttcgagaattgcatatagtaaagactttgaaactctcaaagttg<br>attttcttagcaagctacctgaaatgctgaaaatgttcgaagatcgtttatgtcataaaacatattt<br>aaatggtgatcatgtaacccatcctgacttcatgttgtatgacgctcttgatgttgttttatacatgg<br>acccaatgtgcctggatgcgttcccaaaattagtttgttttaaaaaacgtattgaagctatcccac<br>aaattgataagtacttgaaatccagcaagtatatagcatggcctttgcagggctggcaagcca<br>cgtttggtggtggcgaccatcctccaaaatcggatggttcaactagtctggaagttctgttccag<br>gggcccatggaagccagcccagcatccgggccccgccacttgatggatccacacatcttca<br>cttccaactttaacaattttattggacgccgccagacctacctgtgctacgaagtggagcgcct<br>ggacaatggcacctcggtcaagatggaccagcacaggggctttctacacaaccaggctaag<br>aatcttctctgtggcttttacggccgccatgcggagctgcgcttcttggacctggttccttctttgc<br>agttggacccggcccagatctacagggtcacttggttcatctcctggagcccctgcttctcctg<br>gggctgtgccggggaagtgcgtgcgttccttcaggagaacacacacgtgagactgcgtatct<br>tcgctgcccgcatctatgattacgacaccgattataaggaggcactgcaaatgctgcgggatg<br>ctggggcccaagtctccatcatgacctacgatgaatttaagcactgctgggacacctttgtgga<br>ccaccagggatgtcccttccagccctgggatggactggatgagcacagccaagccctgagt<br>gggaggctgcgggccattctccagaatcagggaaacctggaagttctgttccaggggcccct<br>cgagcaccaccaccaccaccaccactaattgattaatacctaggctgctaaacaaagcc<br>cgaaaggaagctgagttggctgctgccaccgctgagcaataactagcataaccccttggggc<br>ctctaaacgggtcttgaggggttttttgctgaaaggaggaactatatccggat

Note: the red part of the sequence is the coding sequence for eA3A-v10.

Table S9. Sequence information of the amino acid composition of the eA3A-v10 protein.

Name	amino acid composition
eA3A-v10 protein	MEASPASGPRHLMDPHIFTSNFNNFIGRRQTYLCYEVERLD NGTSVKMDQHRGFLHNQAKNLLCGFYGRHAELRFLDLVP SLQLDPAQIYRVTWFISWSPCFSWGCAGEVRAFLQENTHV RLRIFAARIYDYDTDYKEALQMLRDAGAQVSIMTYDEFKH CWDTFVDHQGCPFQPWDGLDEHSQALSGRLRAILQNQGN

Figure S1. Characterization of the deaminase selectivity of wtA3A toward C, 5mC and 5hmC in different sequence context by Sanger sequencing. DNA-C, DNA-5mC and DNA-5hmC were used for the evaluation. (A) The amino acid composition of wtA3A. (B) The sequencing results of DNA-C, DNA-5mC, and DNA-5hmC after wtA3A treatment. All the C in DNA-C and all the 5mC in DNA-5mC were deaminated and read as T; 5hmC in DNA-5hmC were partially deaminated and therefore were partially read as C and partially read as T.

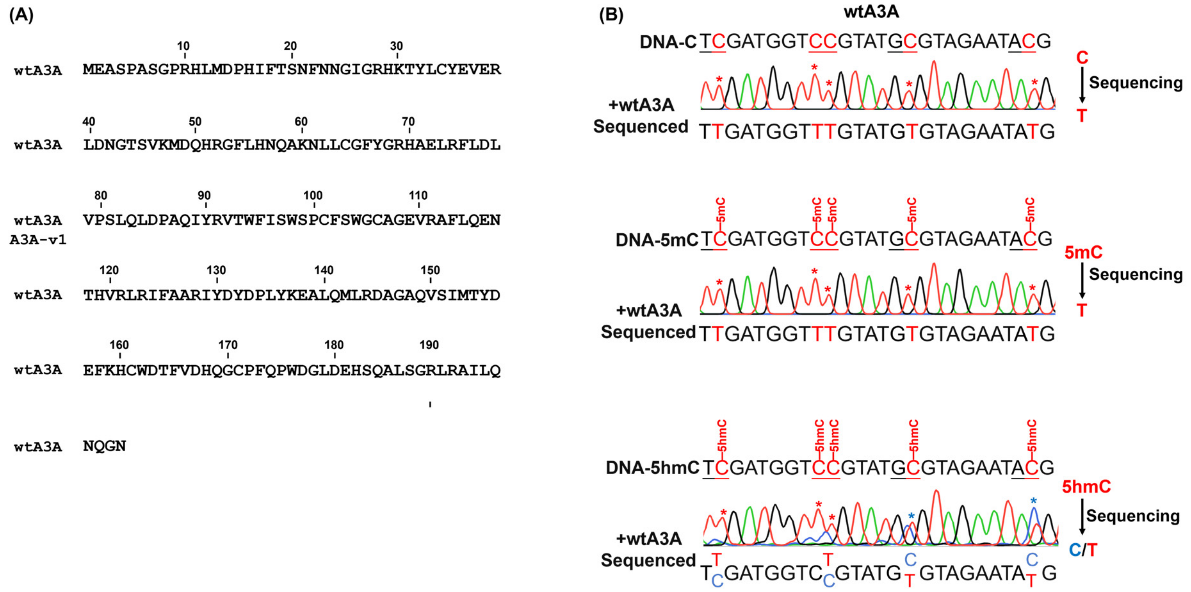

Figure S2. Principle of the engineered deaminase-mediated sequencing (EDM-seq). (A) In the eA3A-5 treatment, the enzyme deaminates both C and 5mC in DNA, causing them to be read as T during sequencing. However, 5hmC in RC (GC and AC) sites is resistant to deamination and is read as C during sequencing. 5hmC in YC (TC and CC) sites is partially deaminated, resulting in a combination of C and T readouts during sequencing. This means that after eA3A-5 treatment, only 5hmC in RC sites can be accurately detected. (B) In the eA3A-9 treatment, all C in DNA are deaminated and read as T during sequencing. 5mC in YC (TC and CC) sites are also deaminated and read as T during sequencing. However, 5mC in RC (GC and AC) sites is only partially deaminated, leading to a mix of C and T readouts during sequencing. Additionally, all 5hmC sites in DNA are resistant to deamination and are read as C during sequencing. Therefore, after eA3A-9 treatment, only 5hmC in YC sites can be precisely detected. In this context, "R" represents "G" and "A", while "Y" represents "T" and "C". Overall, the sequencing readouts can be quite complex and require careful interpretation.

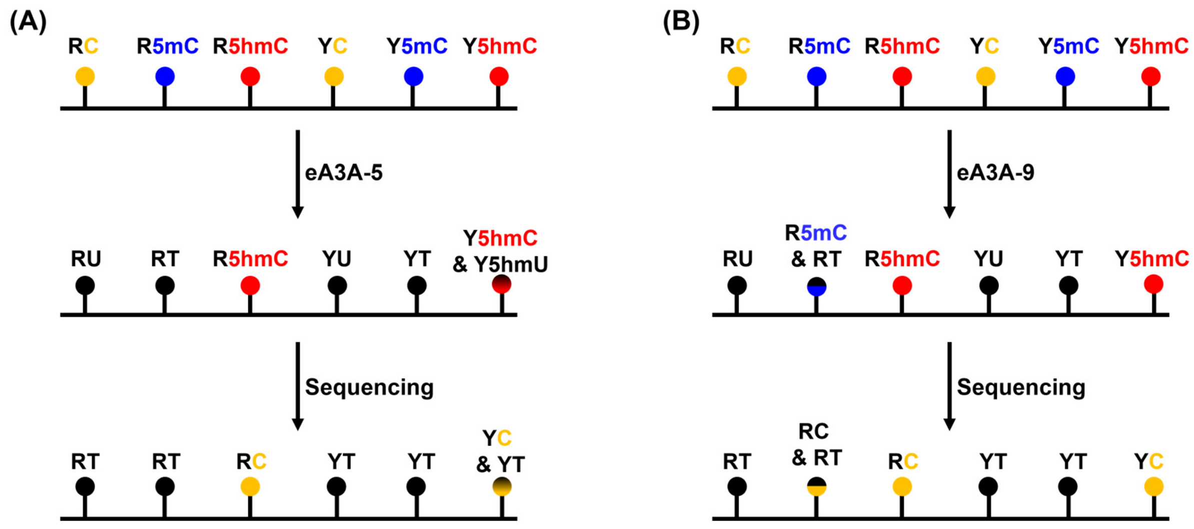

Figure S3. Characterization of the deaminase selectivity of eA3A-v1 toward C, 5mC and 5hmC in different sequence context by Sanger sequencing. DNA-C, DNA-5mC and DNA-5hmC were used for the evaluation. (A) The amino acid composition of wtA3A and eA3A-v1 proteins. (B) The sequencing results of DNA-C, DNA-5mC, and DNA-5hmC after eA3A-v1 treatment. All the C in DNA-C and all the 5mC in DNA-5mC were deaminated and read as T; 5hmC in TC and CC sites were partially deaminated and therefore were partially read as C and partially read as T; 5hmC in GC and AC sites were resistant to deamination and were still read as C.

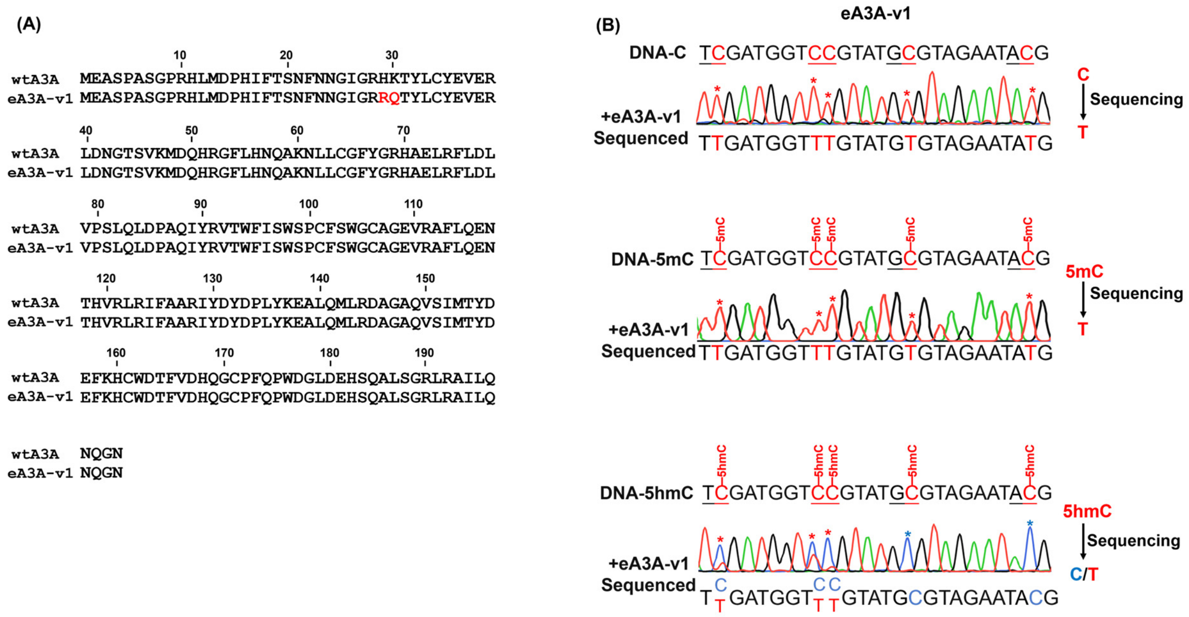

Figure S4. Characterization of the deaminase selectivity of eA3A-v2 toward C, 5mC and 5hmC in different sequence context by Sanger sequencing. DNA-C, DNA-5mC and DNA-5hmC were used for the evaluation. (A) The amino acid composition of wtA3A and eA3A-v2 proteins. (B) The sequencing results of DNA-C, DNA-5mC, and DNA-5hmC after eA3A-v2 treatment. All the C were deaminated and read as T; 5mC were partially deaminated and therefore were partially read as C and partially read as T; 5hmC were resistant to deamination and were still read as C.

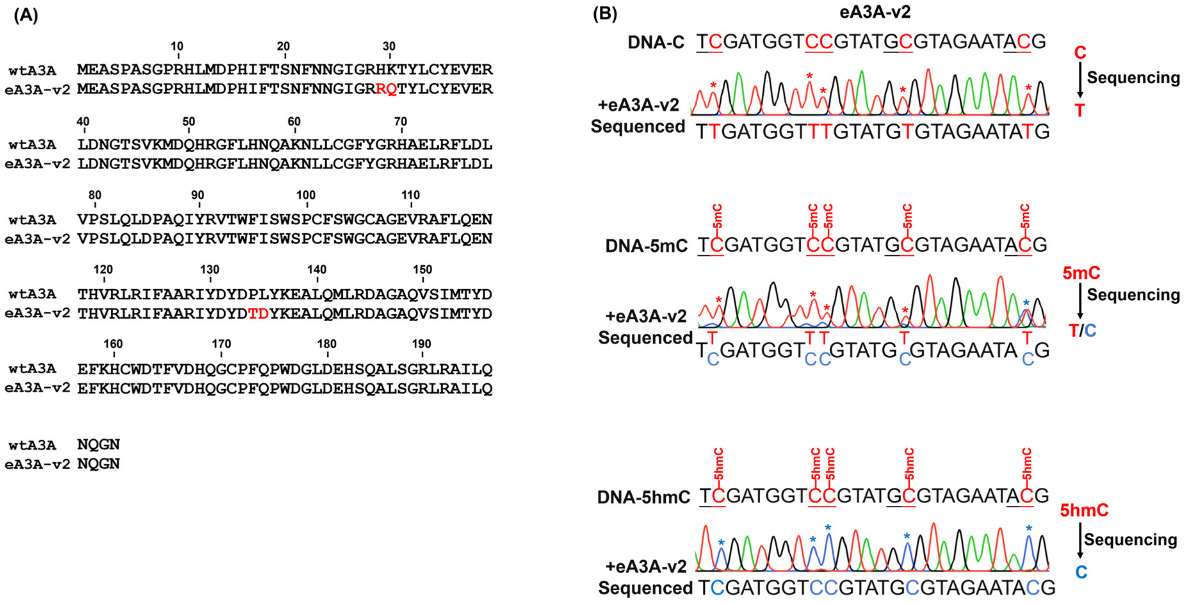

Figure S5. Characterization of the deaminase selectivity of eA3A-v3 toward C, 5mC and 5hmC in different sequence context by Sanger sequencing. DNA-C, DNA-5mC and DNA-5hmC were used for the evaluation. (A) The amino acid composition of wtA3A and eA3A-v3 proteins. (B) The sequencing results of DNA-C, DNA-5mC, and DNA-5hmC after eA3A-v3 treatment. All the C were deaminated and read as T; 5mC were partially deaminated and therefore were partially read as C and partially read as T; 5hmC were resistant to deamination and were still read as C.

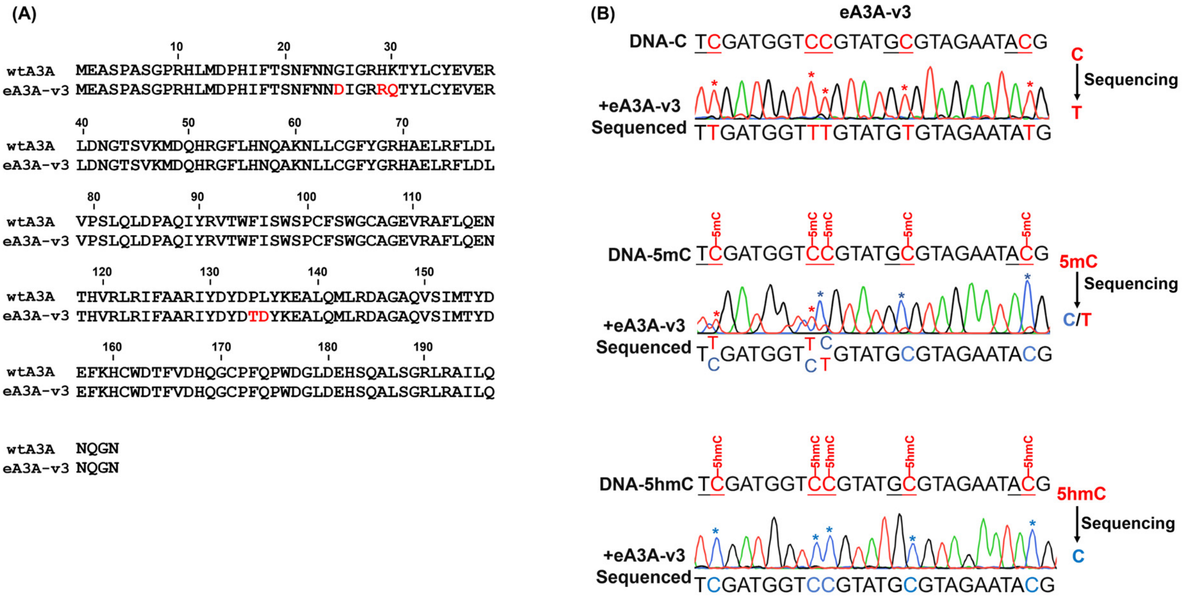

Figure S6. Characterization of the deaminase selectivity of eA3A-v4 toward C, 5mC and 5hmC in different sequence context by Sanger sequencing. DNA-C, DNA-5mC and DNA-5hmC were used for the evaluation. (A) The amino acid composition of wtA3A and eA3A-v4 proteins. (B) The sequencing results of DNA-C, DNA-5mC, and DNA-5hmC after eA3A-v4 treatment. All the C and 5mC were deaminated and read as T; 5hmC in TC and CC sites were deaminated and read as T; 5hmC in GC and AC sites were partially deaminated and therefore were partially read as C and partially read as T.

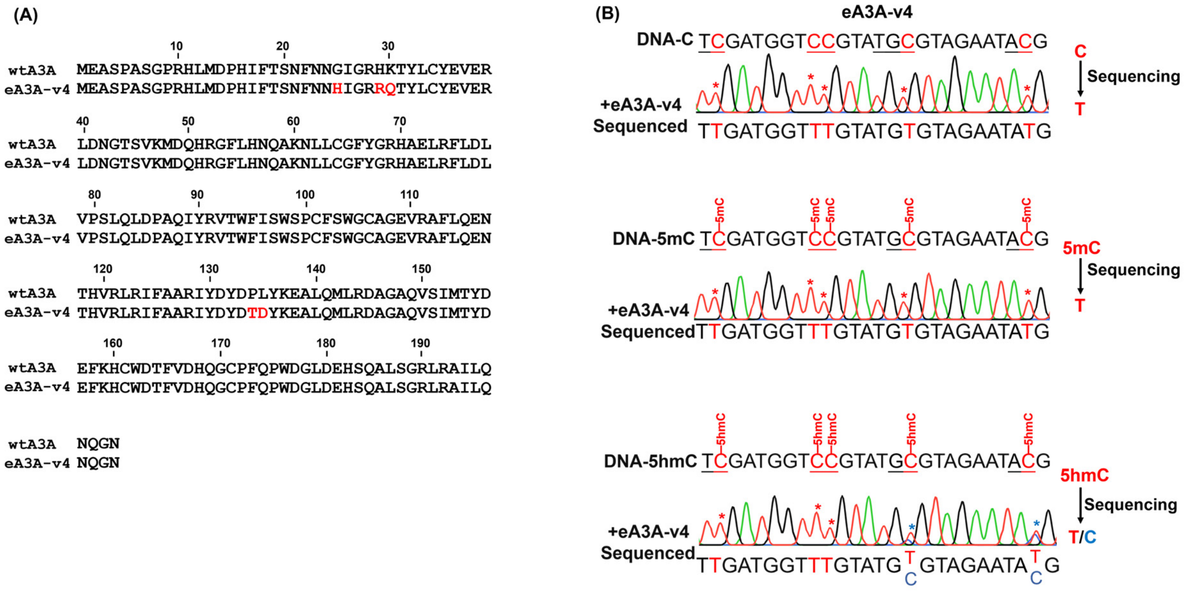

Figure S7. Characterization of the deaminase selectivity of eA3A-v5 toward C, 5mC and 5hmC in different sequence context by Sanger sequencing. DNA-C, DNA-5mC and DNA-5hmC were used for the evaluation. (A) The amino acid composition of wtA3A and eA3A-v5 proteins. (B) The sequencing results of DNA-C, DNA-5mC, and DNA-5hmC after eA3A-v5 treatment. All the C in DNA-C and all the 5mC in DNA-5mC were deaminated and read as T; 5hmC in TC and CC sites were partially deaminated and therefore were partially read as C and partially read as T; 5hmC in GC and AC sites were resistant to deamination and were still read as C.

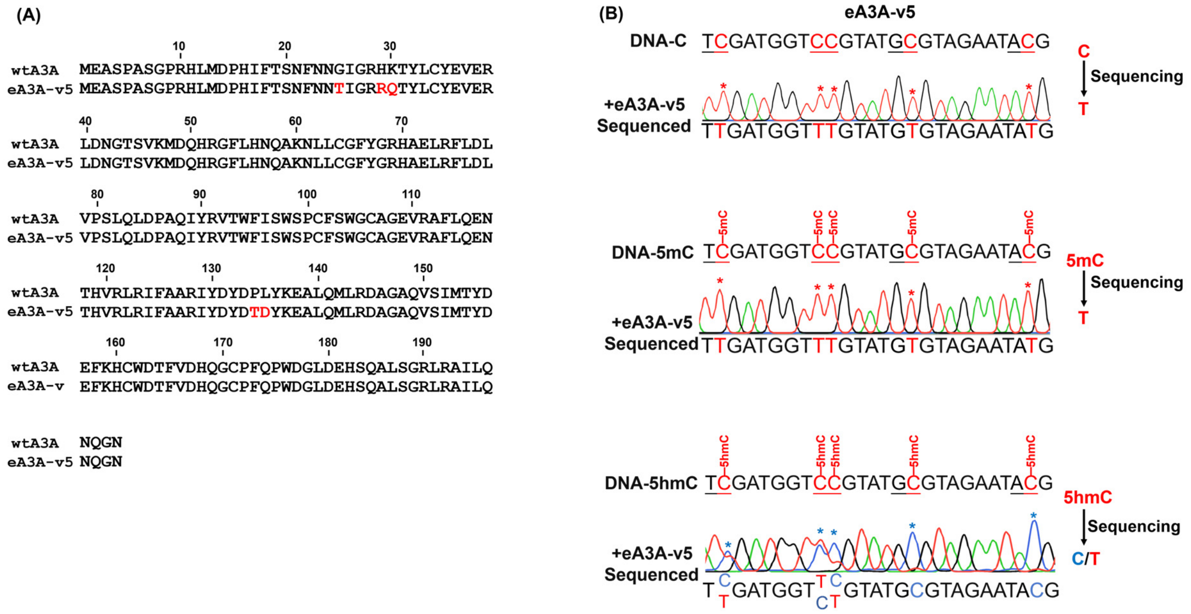

Figure S8. Characterization of the deaminase selectivity of eA3A-v6 toward C, 5mC and 5hmC in different sequence context by Sanger sequencing. DNA-C, DNA-5mC and DNA-5hmC were used for the evaluation. (A) The amino acid composition of wtA3A and eA3A-v6 proteins. (B) The sequencing results of DNA-C, DNA-5mC, and DNA-5hmC after eA3A-v6 treatment. All the C in DNA-C and all the 5mC in DNA-5mC were deaminated and read as T; all the 5hmC in DNA-5hmC were partially deaminated and therefore were partially read as C and partially read as T.

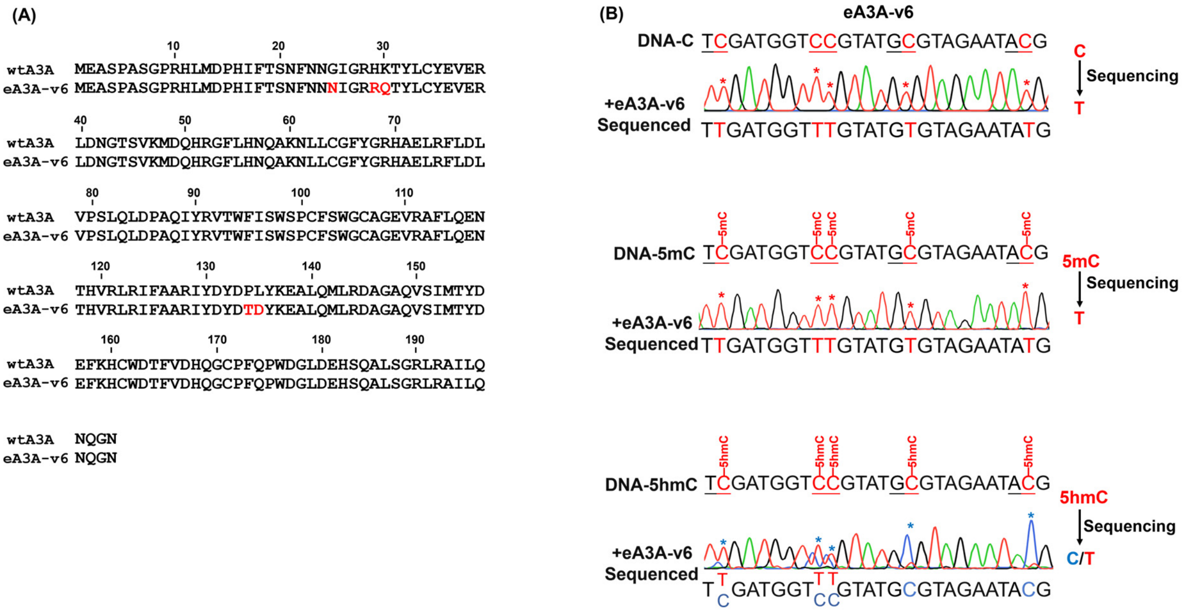

Figure S9. Characterization of the deaminase selectivity of eA3A-v7 toward C, 5mC and 5hmC in different sequence context by Sanger sequencing. DNA-C, DNA-5mC and DNA-5hmC were used for the evaluation. (A) The amino acid composition of wtA3A and eA3A-v7 proteins. (B) The sequencing results of DNA-C, DNA-5mC, and DNA-5hmC after eA3A-v7 treatment. All the C in DNA-C and all the 5mC in DNA-5mC were deaminated and read as T; 5hmC in TC and CC sites were partially deaminated and therefore were partially read as C and partially read as T; 5hmC in GC and AC sites resistant to deamination and were still read as C.

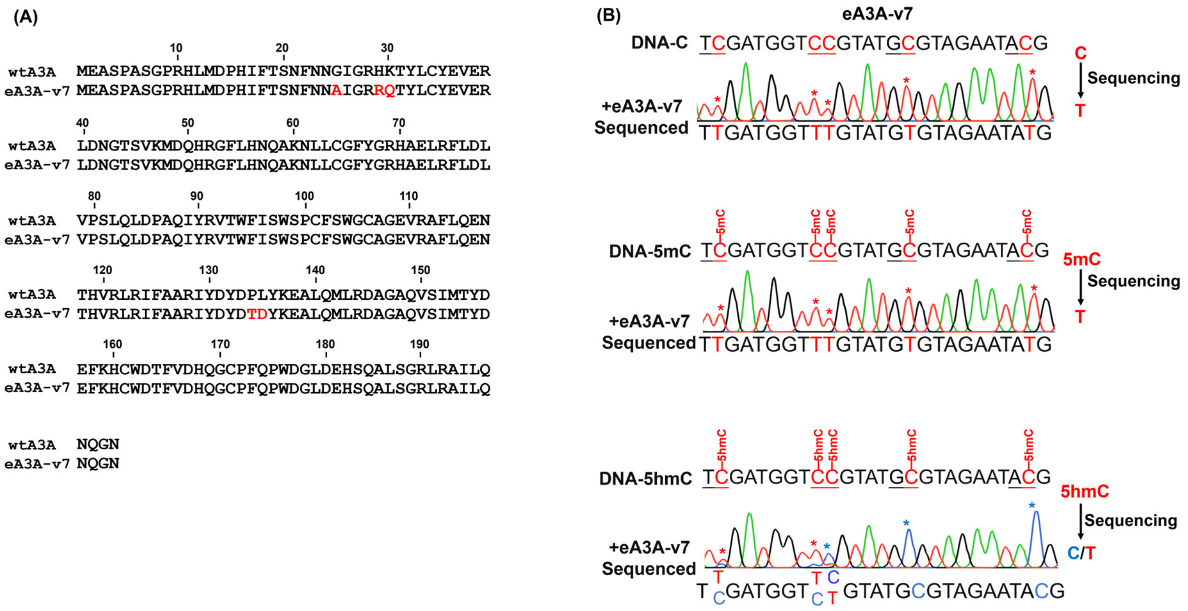

Figure S10. Characterization of the deaminase selectivity of eA3A-v8 toward C, 5mC and 5hmC in different sequence context by Sanger sequencing. DNA-C, DNA-5mC and DNA-5hmC were used for the evaluation. (A) The amino acid composition of wtA3A and eA3A-v8 proteins. (B) The sequencing results of DNA-C, DNA-5mC, and DNA-5hmC after eA3A-v8 treatment. All the C in DNA-C and all the 5mC in DNA-5mC were deaminated and read as T; 5hmC in TC and CC sites were partially deaminated and therefore were partially read as C and partially read as T; 5hmC in GC and AC sites were resistant to deamination and were still read as C.

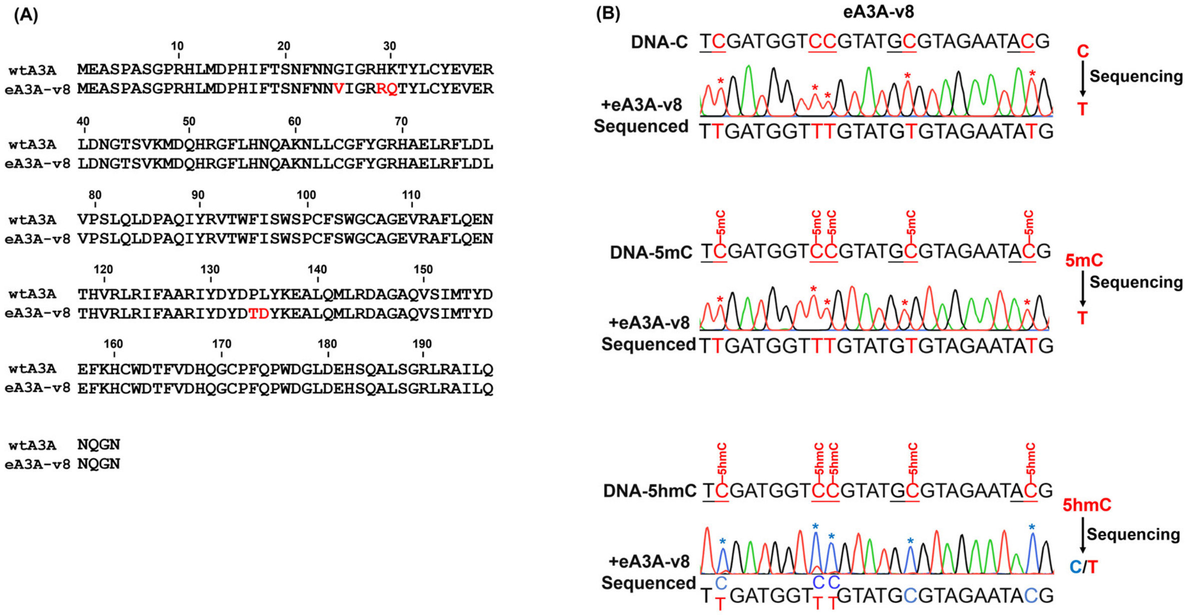

Figure S11. Characterization of the deaminase selectivity of eA3A-v9 toward C, 5mC and 5hmC in different sequence context by Sanger sequencing. DNA-C, DNA-5mC and DNA-5hmC were used for the evaluation. (A) The amino acid composition of wtA3A and eA3A-v9 proteins. (B) The sequencing results of DNA-C, DNA-5mC, and DNA-5hmC after eA3A-v9 treatment. All the C in DNA-C and the 5mC in TC and CC sites were deaminated and read as T; 5mC in GC and AC sites were partially deaminated and therefore were partially read as C and partially read as T; 5hmC in DNA-5hmC were resistant to deamination and were still read as C.

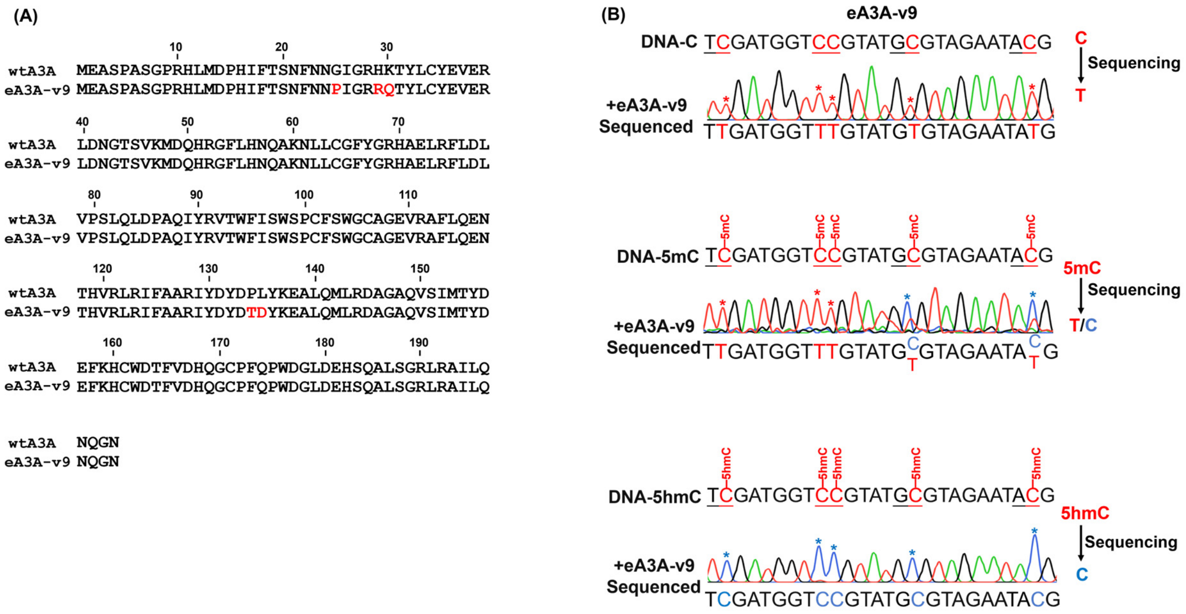

Figure S12. The amino acid composition of wtA3A and eA3A-v10 proteins.

```
                      10        20        30
wtA3A     MEASPASGPRHLMDPHIFTSNFNNGIGRHKTYLCYEVER
eA3A-v10  MEASPASGPRHLMDPHIFTSNFNNFIGRRQTYLCYEVER

          40        50        60        70
wtA3A     LDNGTSVKMDQHRGFLHNQAKNLLCGFYGRHAELRFLDL
eA3A-v10  LDNGTSVKMDQHRGFLHNQAKNLLCGFYGRHAELRFLDL

           80        90        100       110
wtA3A     VPSLQLDPAQIYRVTWFISWSPCFSWGCAGEVRAFLQEN
eA3A-v10  VPSLQLDPAQIYRVTWFISWSPCFSWGCAGEVRAFLQEN

           120       130       140       150
wtA3A     THVRLRIFAARIYDYDPLYKEALQMLRDAGAQVSIMTYD
eA3A-v10  THVRLRIFAARIYDYDTDYKEALQMLRDAGAQVSIMTYD

            160       170       180      190
wtA3A     EFKHCWDTFVDHQGCPFQPWDGLDEHSQALSGRLRAILQ
eA3A-v10  EFKHCWDTFVDHQGCPFQPWDGLDEHSQALSGRLRAILQ

wtA3A     NQGN
eA3A-v10  NQGN
```

Figure S13. Extracted-ion chromatograms of dC, 5mC, 5hmC, dA, dG and dT from wtA3A-treated and untreated DNA by LC-MS/MS analysis. C-containing DNA mixture (TC-C, CC-C, AC-C and GC-C), 5mC-containing DNA mixture (TC-5mC, CC-5mC, AC-5mC and GC-5mC) and 5hmC-containing DNA mixture (TC-5hmC, CC-5hmC, AC-5hmC and GC-5hmC) were used for the evaluation. (A) Extracted-ion chromatograms of dC, 5mC and 5hmC from C-containing DNA mixture, 5mC-containing DNA mixture and 5mC-containing DNA mixture without or with wtA3A treatment. (B) Extracted-ion chromatograms of dA, dG and dT from C-containing DNA mixture without or with wtA3A treatment.

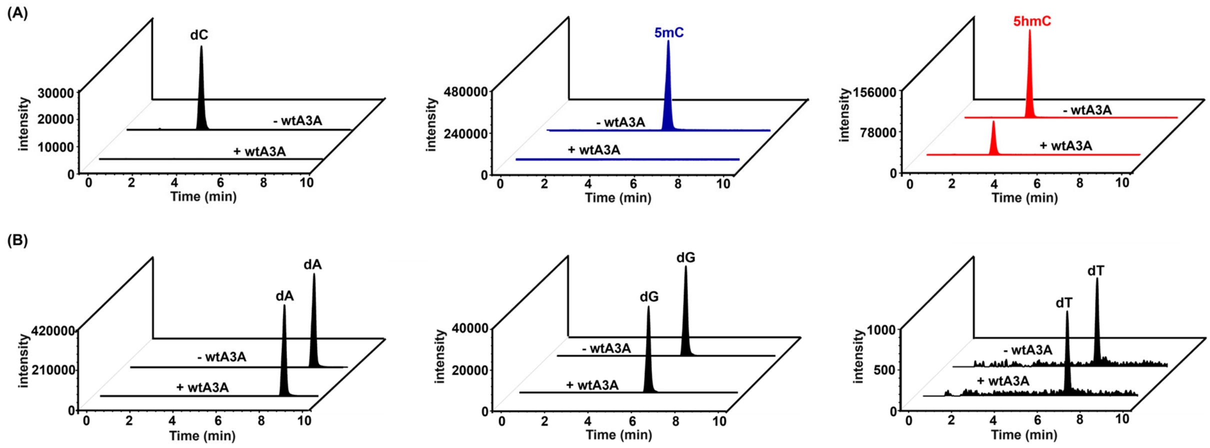

Figure S14. Quantitative evaluation of the deamination activity of wtA3A toward C, 5mC and 5hmC by steady-state kinetics analysis. C-containing DNA mixture (TC-C, CC-C, AC-C and GC-C), 5mC-containing DNA mixture (TC-5mC, CC-5mC, AC-5mC and GC-5mC) and 5hmC-containing DNA mixture (TC-5hmC, CC-5hmC, AC-5hmC and GC-5hmC) were used for the evaluation. (A) Kinetic constants of wtA3A acting on C, 5mC and 5hmC. (B)-(D) Rate versus substrate concentration curves of the substrates of C-containing DNA mixture, 5mC-containing DNA mixture and 5hmC-containing DNA mixture.

(A)

Substrate	k_{cat} (min^{-1})	K_M (μM)	k_{cat}/K_M ($\mu M^{-1} min^{-1}$)	Relative activity
C	54.49 ± 3.46	0.60 ± 0.03	90.82	1.0
5mC	24.02 ± 3.93	1.07 ± 0.11	22.45	0.25
5hmC	0.02 ± 0.003	0.06 ± 0.06	0.34	3.6×10^{-3}

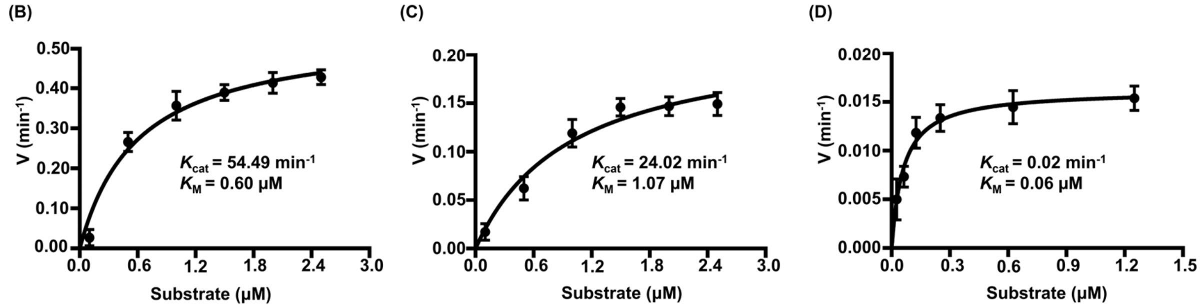

Figure S15. Agarose gel electrophoresis analysis of the PCR products from different amounts of DNA treated with eA3A-v10. (A) 100 ng, 1 ng and 1 pg of DNA-C were treated with eA3A-v10, respectively, followed by PCR amplification. (B) 1 pg of DNA-C, DNA-5mC and DNA-5hmC were treated with eA3A-v10, respectively, followed by PCR amplification. “M” represents DNA marker.

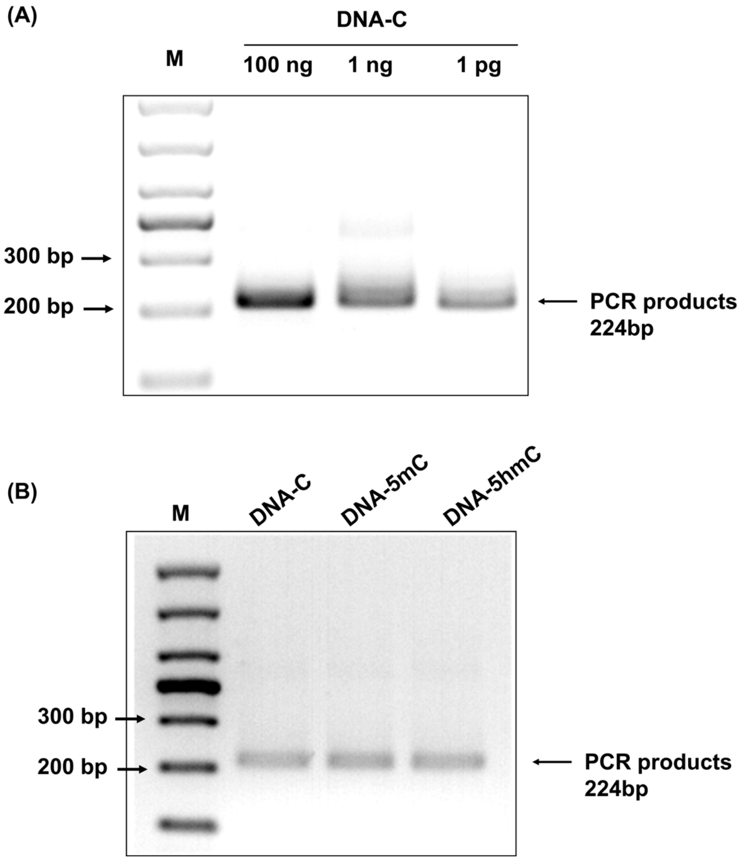

Figure S16. Evaluation of the detection capability of the SSD-seq with small amount of DNA. 1 pg of DNA-C, DNA-5mC and DNA-5hmC were subjected to SSD-seq analysis. the Sanger sequencing results showed that all the C in DNA-C and all the 5mC in DNA-5mC were deaminated and read as T. However, all the 5hmC sites in DNA-5hmC were not deaminated and still read as C.

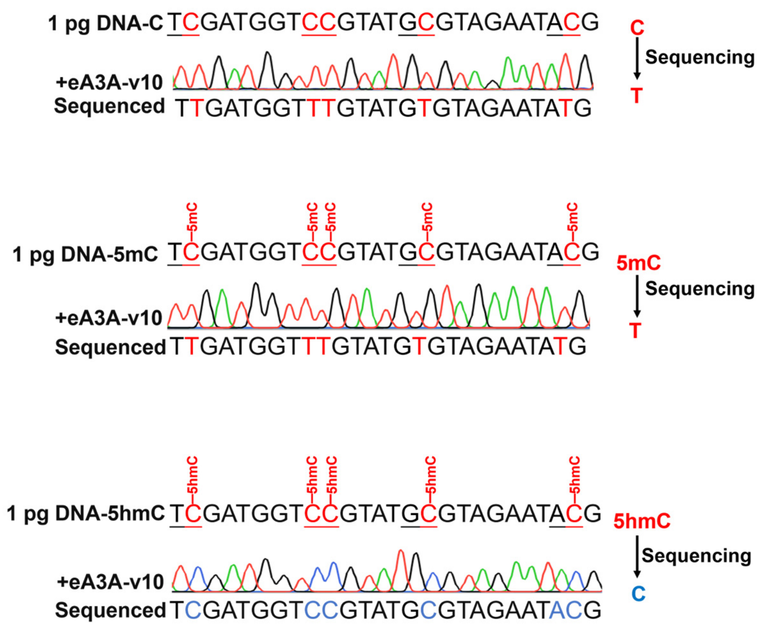

Figure S17. Evaluation of the deaminase activity of eA3A-v10 toward C by colony sequencing. the 367-bp DNA-L-C was denatured to ssDNA and then treated with eA3A-v10 followed by colony sequencing. Ten clones for each sample were randomly picked up and sequenced. Three replicates were carried out for the evaluation. A total of 1259 cytosines out of 1260 (totally 42 cytosines/strand × 30 clones = 1260 cytosines) in 367-bp DNA-L-C were read as T. The conversion rate of C-to-T was calculated to be 99.92% (1259/1260 × 100% = 99.92%). Red, read as C. Blue, read as T.

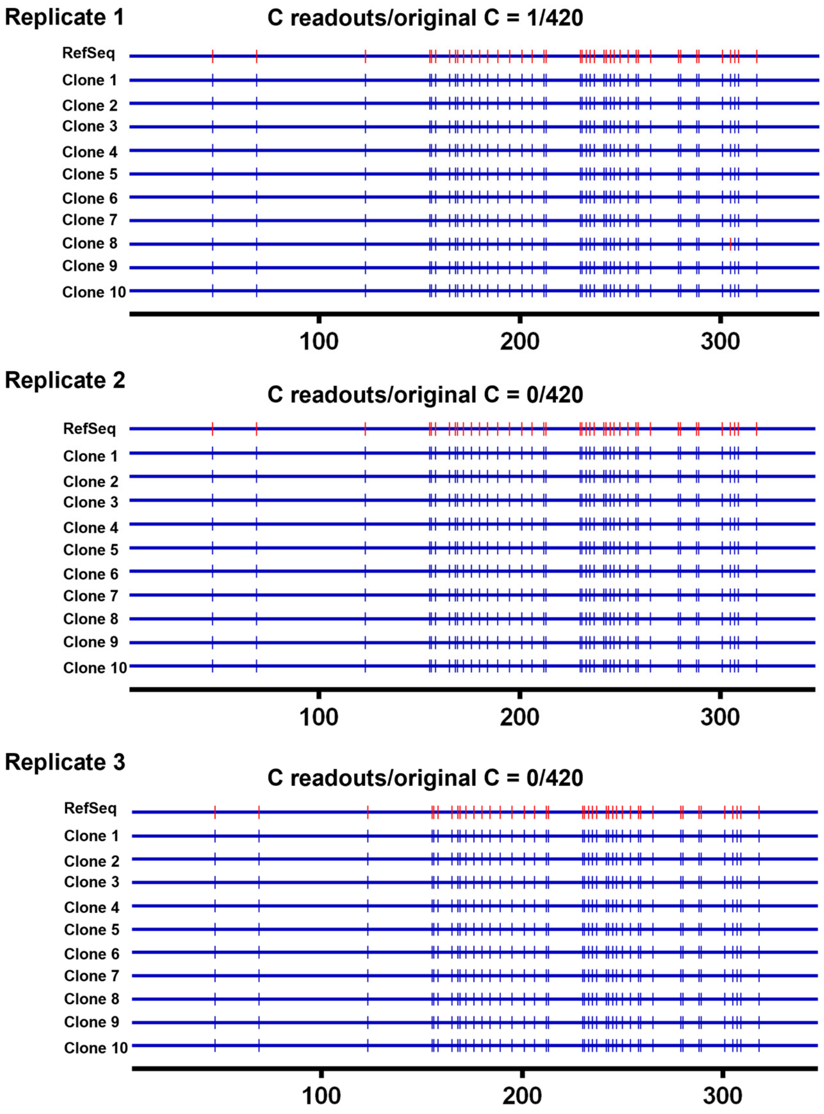

Figure S18. Evaluation of the deaminase activity of eA3A-v10 toward 5mC by colony sequencing. The 367-bp DNA-L-5mC was denatured to ssDNA and then treated with eA3A-v10 followed by colony sequencing. Ten clones for each sample were randomly picked up and sequenced. Three replicates were carried out for the evaluation. A total of 1254 5mC out of 1260 (totally 42 5mC/strand × 30 clones = 1260 5mC sites) in 367-bp DNA-L-5mC were read as T. The conversion rate of 5mC-to-T was calculated to be 99.52% (1254/1260 × 100% = 99.52%). Red, read as C. Blue, read as T.

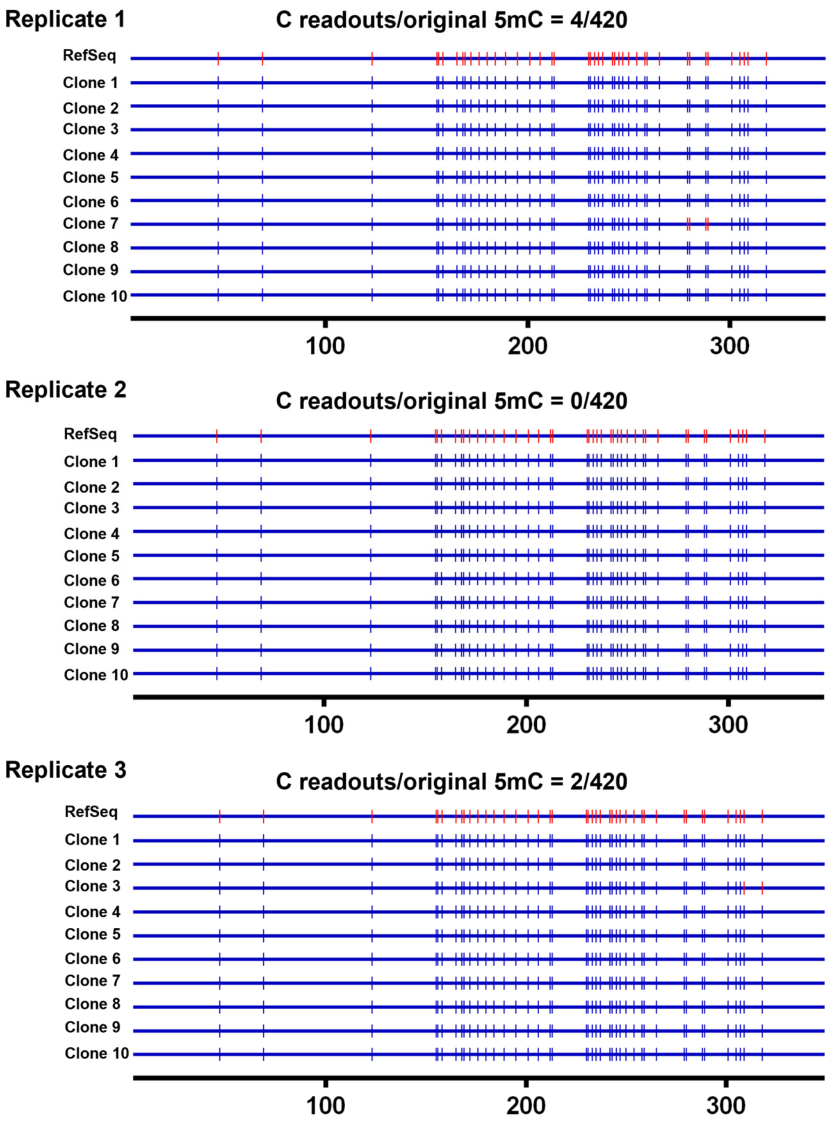

Figure S19. Evaluation of the deaminase activity of eA3A-v10 toward 5hmC by colony sequencing. The 367-bp DNA-L-5hmC was denatured to ssDNA and then treated with eA3A-v10 followed by colony sequencing. Ten clones for each sample were randomly picked up and sequenced. Three replicates were carried out for the evaluation. A total of 2 5hmC out of 1260 (totally 42 5hmC/strand × 30 clones = 1260 5hmC sites) in 367-bp DNA-L-5hmC were read as T. the conversion rate of 5hmC-to-T was calculated to be 0.16% (2/1260 × 100% = 0.16%). Red, read as C. Blue, read as T.

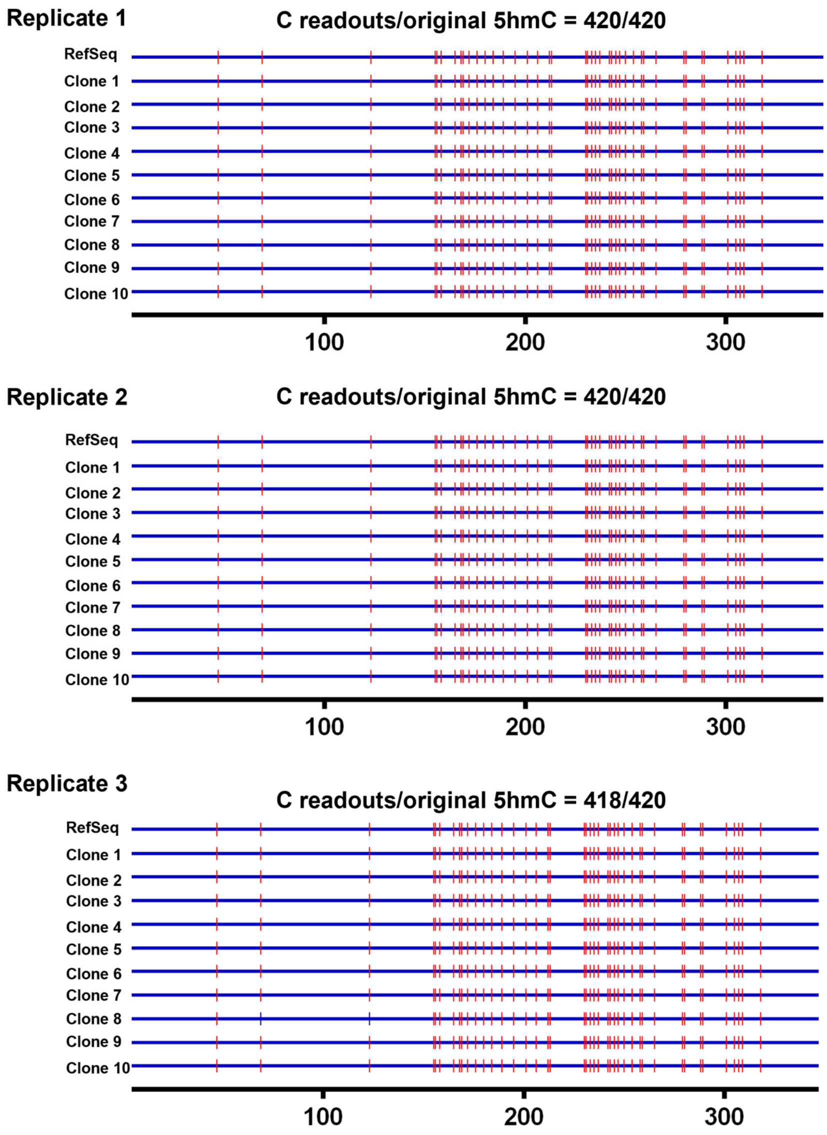

Figure S20. Evaluation of the deaminase activity of wtA3A toward C by colony sequencing. The 367-bp DNA-L-C was denatured to ssDNA and then treated with wtA3A followed by colony sequencing. Ten clones for each sample were randomly picked up and sequenced. Three replicates were carried out for the evaluation. A total of 1260 cytosines out of 1260 (totally 42 cytosines/strand × 30 clones = 1260 cytosines) in 367-bp DNA-L-C were read as T. The conversion rate of C-to-T was calculated to be 100.0% (1260/1260 × 100% = 100.0%). Red, read as C. Blue, read as T.

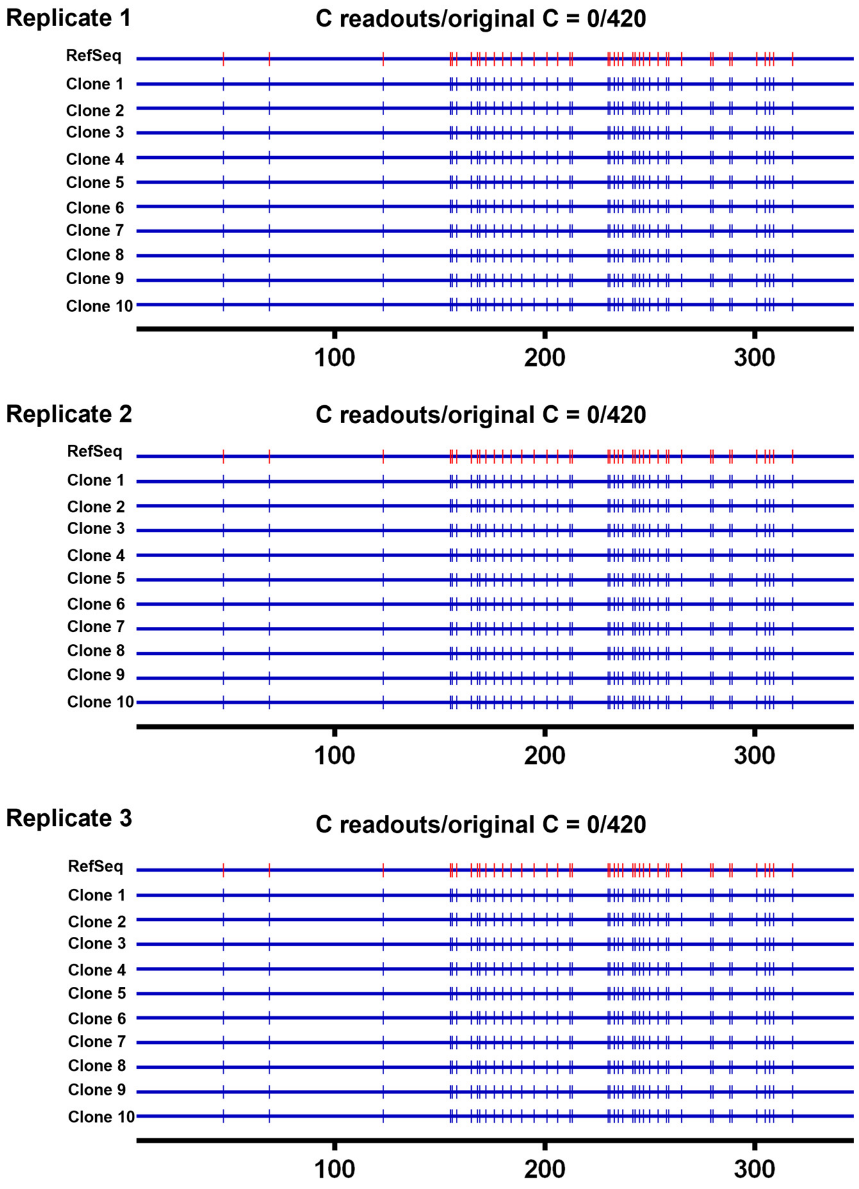

Figure S21. Evaluation of the deaminase activity of wtA3A toward 5mC by colony sequencing. The 367-bp DNA-L-5mC was denatured to ssDNA and then treated with wtA3A followed by colony sequencing. Ten clones for each sample were randomly picked up and sequenced. Three replicates were carried out for the evaluation. A total of 1256 5mC out of 1260 (totally 42 5mC/strand × 30 clones = 1260 5mC sites) in 367-bp DNA-L-5mC were read as T. the conversion rate of 5mC-to-T was calculated to be 99.68% (1256/1260 × 100% = 99.68%). Red, read as C. Blue, read as T.

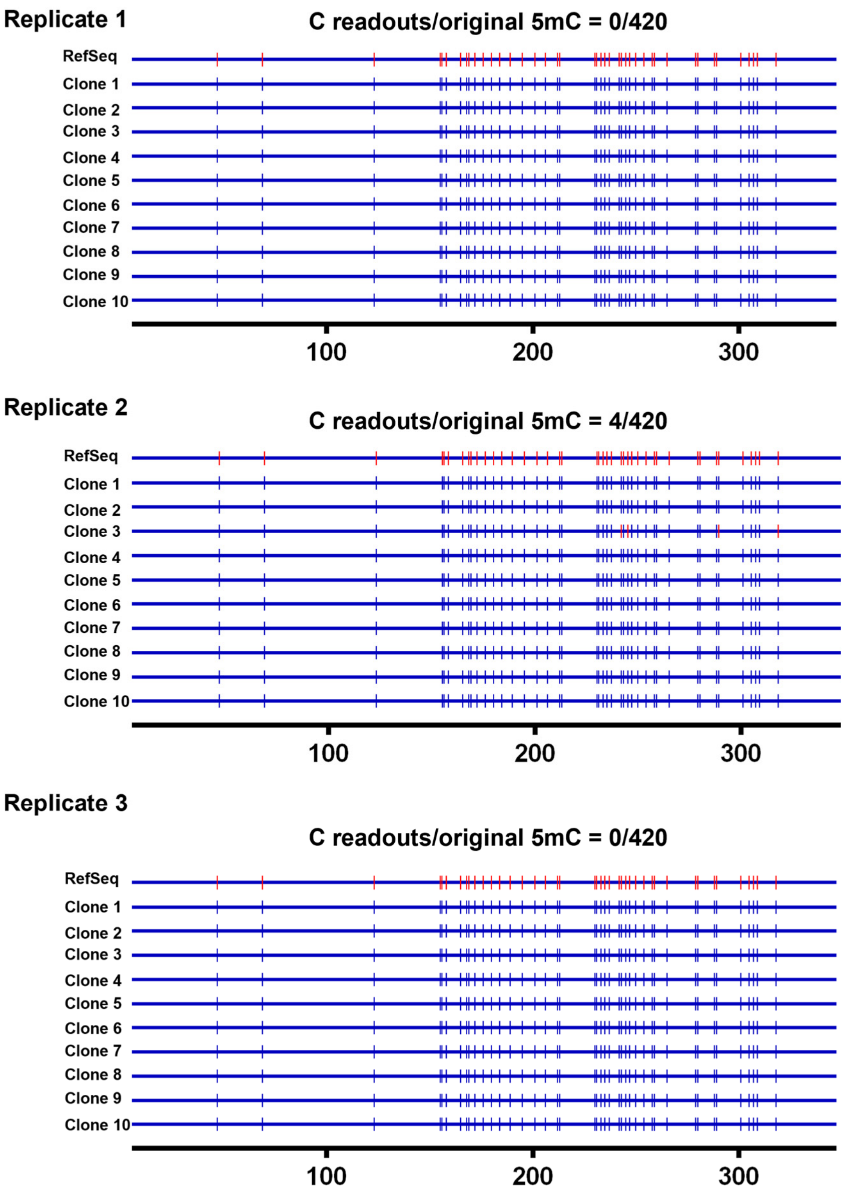

Figure S22. Evaluation of the deaminase activity of wtA3A toward 5hmC by colony sequencing. The 367-bp DNA-L-5hmC was denatured to ssDNA and then treated with wtA3A followed by colony sequencing. Ten clones for each sample were randomly picked up and sequenced. Three replicates were carried out for the evaluation. A total of 1017 5hmC out of 1260 (totally 42 5hmC/strand × 30 clones = 1260 5hmC sites) in 367-bp DNA-L-5hmC were read as T. the conversion rate of 5hmC-to-T was calculated to be 80.71% (1017/1260 × 100% = 80.71%). Red, read as C. Blue, read as T.

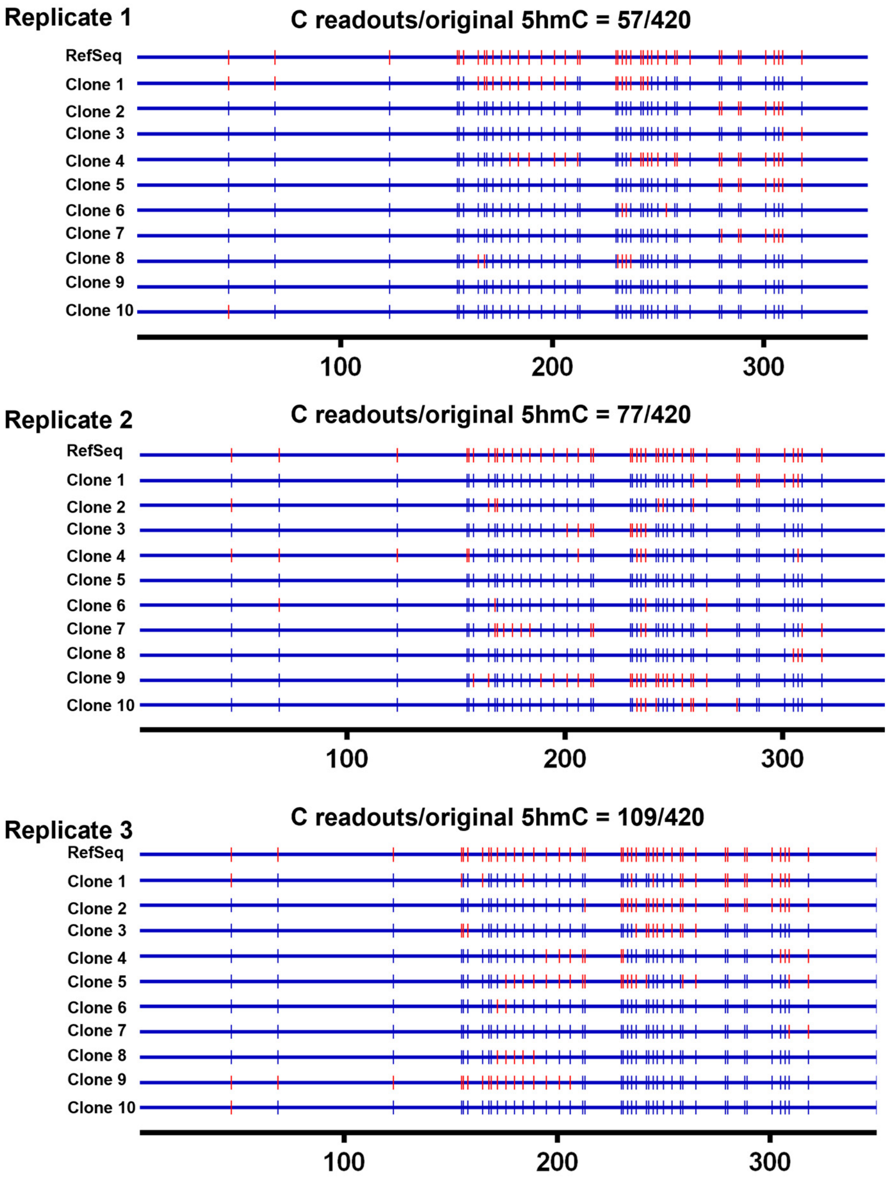

Figure S23. The distribution of 5hmC sites in different chromosomes obtained from SSD-seq and ACE-seq. The numbers in the outer circle represent the chromosomes. The red and green peaks represent the 5hmC sites detected by SSD-seq and ACE-seq, respectively.

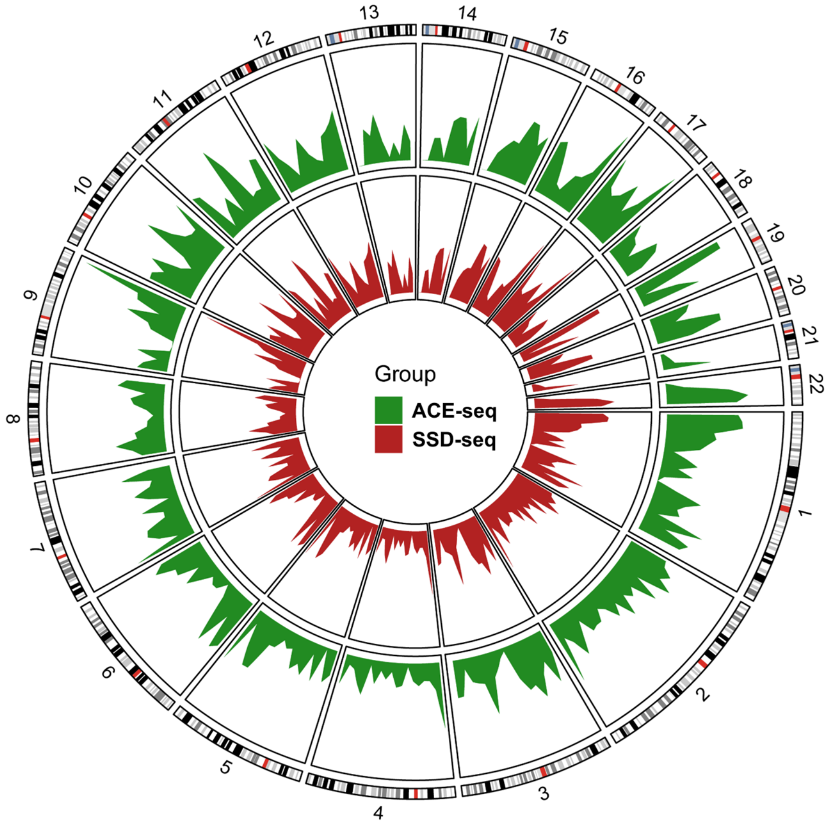

Figure S24. The average 5hmC level around the transcriptional start sites (TSS).

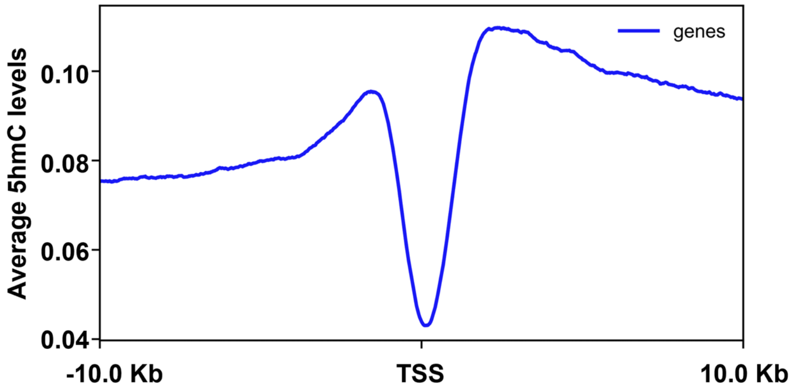

Figure S25. The Gene Ontology and pathway enrichment analysis of 5hmC sites in promotor region. (A) The top 30 most enriched biological processes in the Gene Ontology enrichment analysis of 5hmC sites in promotor region. (B) The top 30 most enriched pathways in the pathway enrichment analysis of 5hmC sites in promotor region.

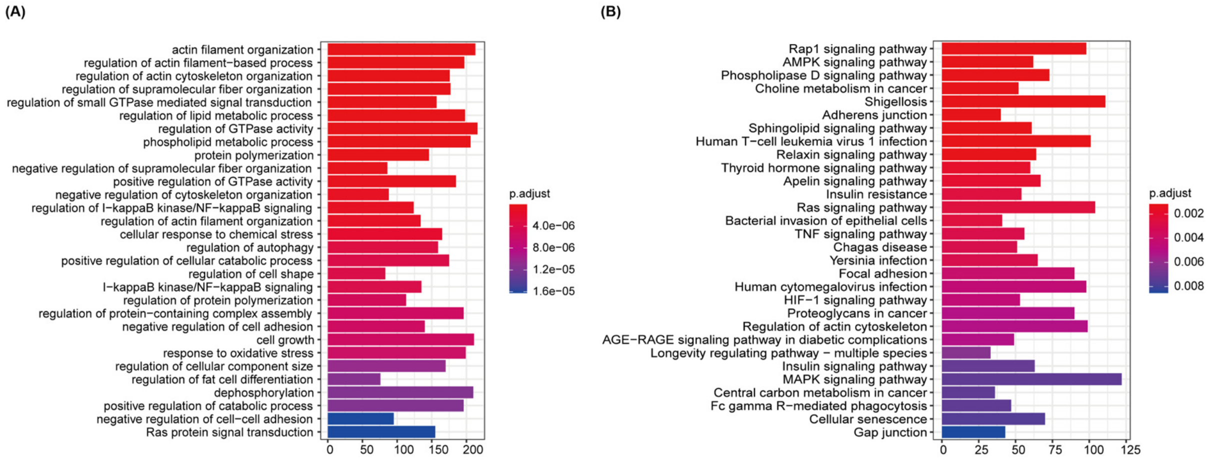

Figure S26. The Gene Ontology and pathway enrichment analysis of 5hmC sites in gene body. (A) The top 30 most enriched biological processes in the Gene Ontology enrichment analysis of 5hmC sites in gene body. (B) The top 30 most enriched pathways in the pathway enrichment analysis of 5hmC sites in gene body.

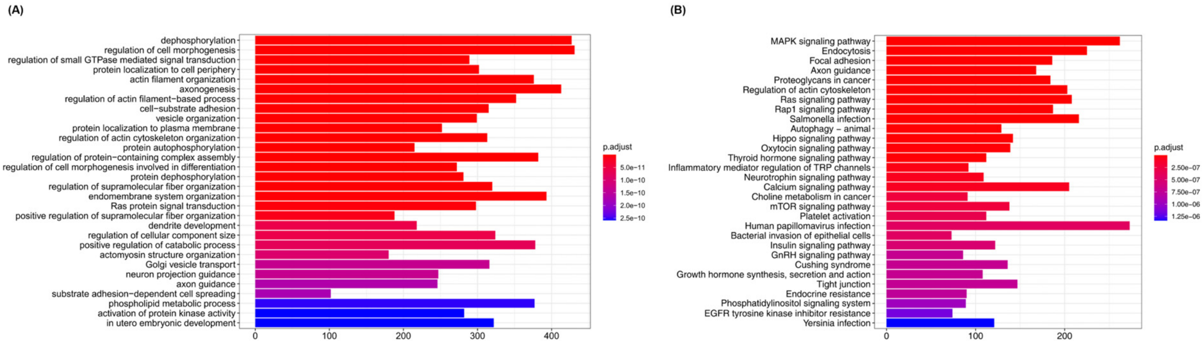

Figure S27. The schematic illustration of plasmid for the expression of wtA3A or eA3A proteins.

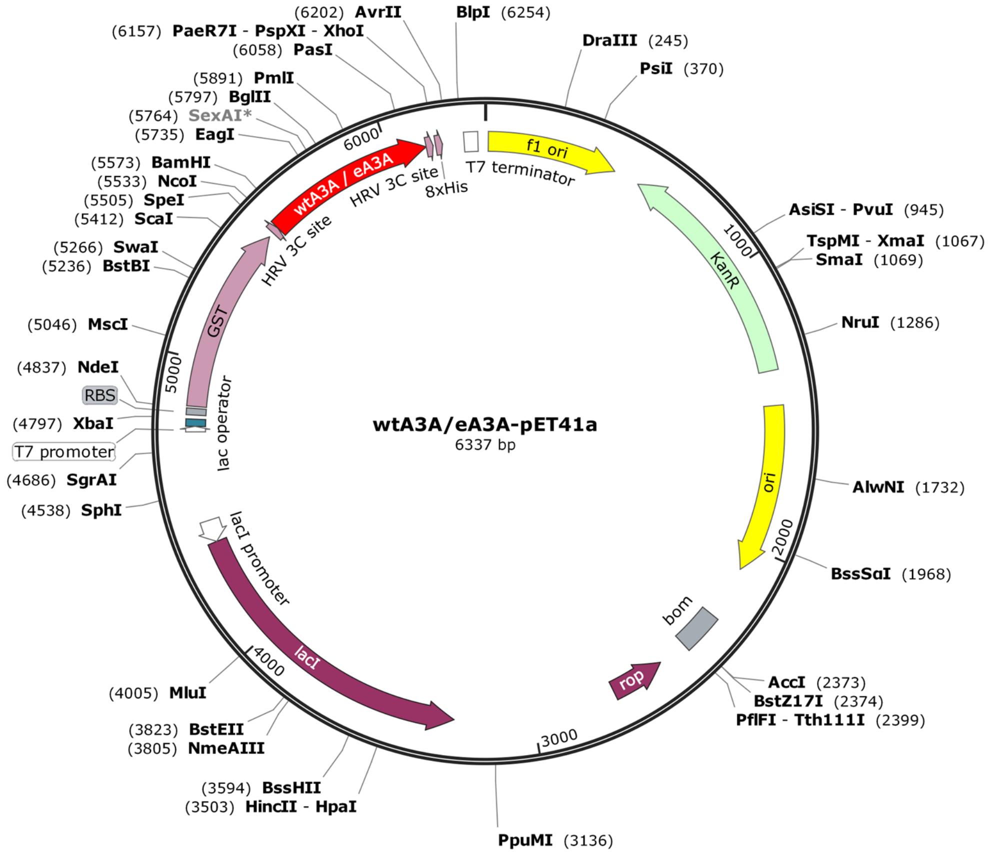

Figure 28. SDS-PAGE analysis of the purified wtA3A and eA3A-v10. (A) SDS-PAGE analysis of the purified wtA3A. (B) SDS-PAGE analysis of the purified eA3A-v10. M, protein marker.

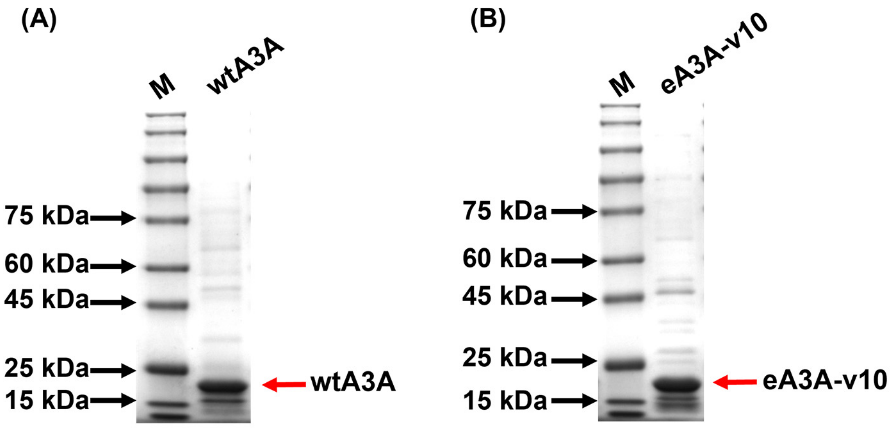

Figure S29. The schematic diagram of library preparation in SSD-seq.

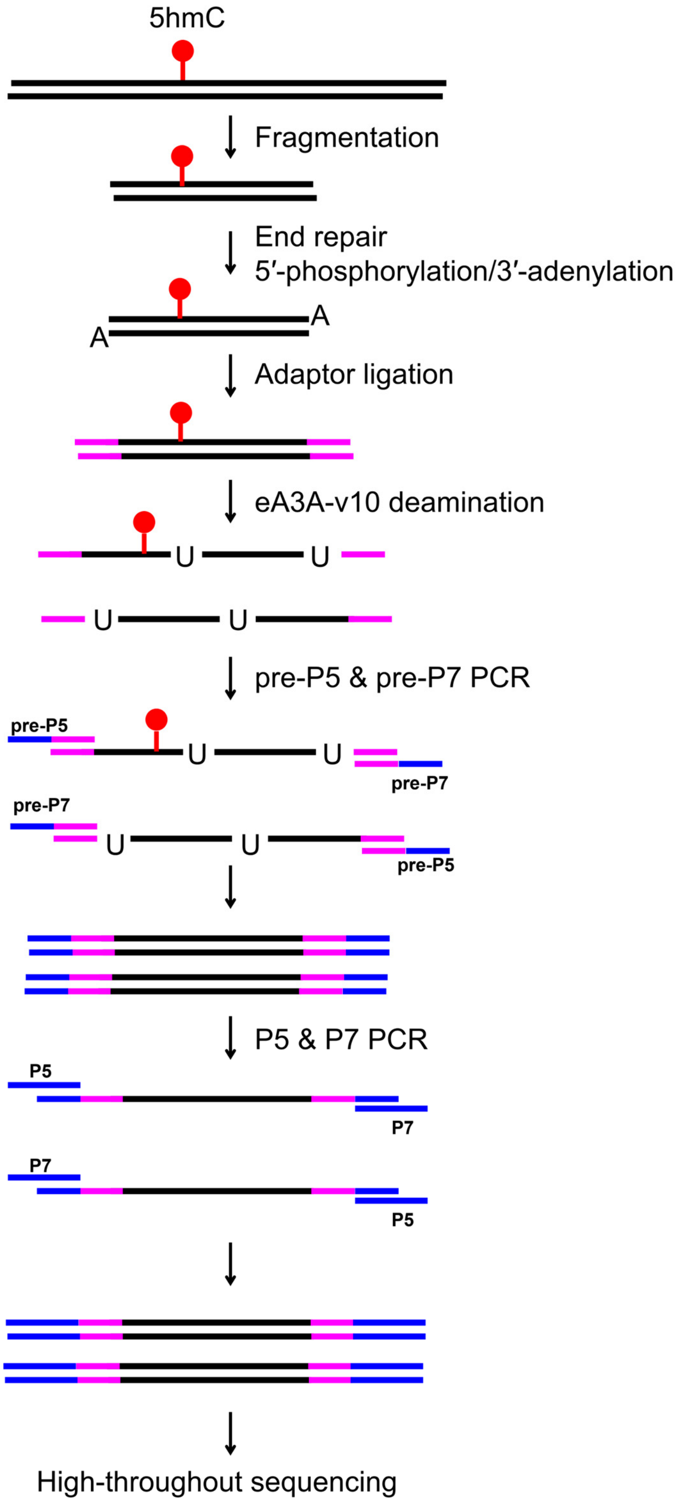

Figure S30. The schematic diagram of library preparation in ACE-seq.

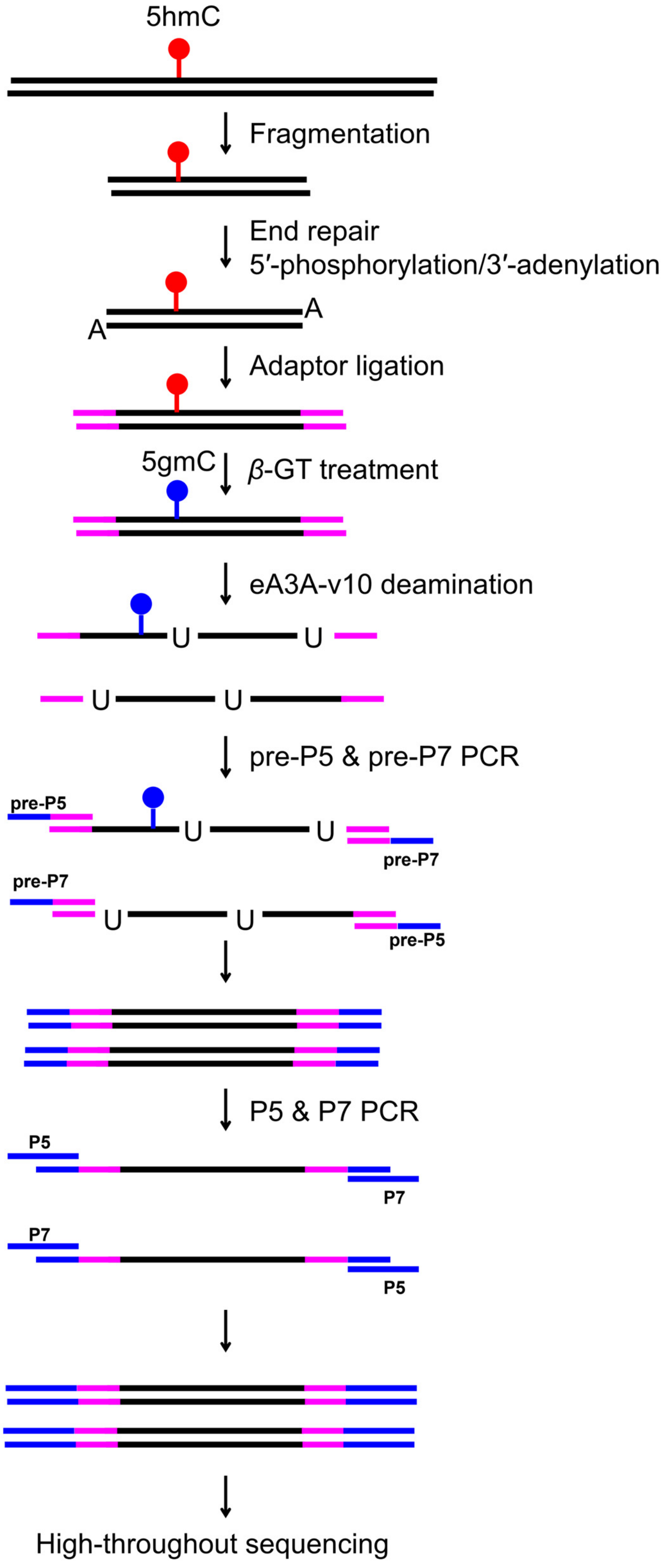

References

1. Li, Q. Y.; Xie, N. B.; Xiong, J.; Yuan, B. F.; Feng, Y. Q., Single-Nucleotide Resolution Analysis of 5-Hydroxymethylcytosine in DNA by Enzyme-Mediated Deamination in Combination with Sequencing. *Anal Chem* **2018,** *90* (24), 14622-14628.
2. Xie, N. B.; Wang, M.; Ji, t. t.; Guo, X.; Ding, J. H.; Yuan, B. F.; Feng, Y. Q., Bisulfite-free and single-nucleotide resolution sequencing of DNA epigenetic modification of 5-hydroxymethylcytosine using engineered deaminase. *Chem Sci* **2022,** *13*, 7046–7056.
